# Does industry take the susceptible subpopulation of asthmatic individuals into consideration when setting derived no‐effect levels?

**DOI:** 10.1002/jat.3352

**Published:** 2016-06-09

**Authors:** Mia K. V. Johansson, Gunnar Johanson, Mattias Öberg, Linda Schenk

**Affiliations:** ^1^ Unit of Work Environment ToxicologyInstitute of Environmental Medicine, Karolinska InstitutetStockholmSweden; ^2^ Swedish Toxicology Sciences Research Center (Swetox)SödertäljeSweden; ^3^ Department of Philosophy and HistoryRoyal Institute of TechnologyStockholmSweden

**Keywords:** asthma, chemicals regulation, policy, risk assessment, guideline values, inhalation, REACH, SCOEL, EU

## Abstract

Asthma, a chronic respiratory disease, can be aggravated by exposure to certain chemical irritants. The objectives were first to investigate the extent to which experimental observations on asthmatic subjects are taken into consideration in connection with the registration process under the EU REACH regulation, and second, to determine whether asthmatics are provided adequate protection by the derived no‐effect levels (DNELs) for acute inhalation exposure. We identified substances for which experimental data on the pulmonary functions of asthmatics exposed to chemicals under controlled conditions are available. The effect concentrations were then compared with DNELs and other guideline and limit values. As of April 2015, only 2.6% of 269 classified irritants had available experimental data on asthmatics. Fourteen of the 22 identified substances with available data were fully registered under REACH and we retrieved 114 reliable studies related to these. Sixty‐three of these studies, involving nine of the 14 substances, were cited by the REACH registrants. However, only 17 of the 114 studies, involving four substances, were regarded as key studies. Furthermore, many of the DNELs for acute inhalation were higher than estimated effect levels for asthmatics, i.e., lowest observed adverse effect concentrations or no‐observed adverse effect concentrations, indicating low or no safety margin. We conclude that REACH registrants tend to disregard findings on asthmatics when deriving these DNELs. In addition, we found examples of DNELs, particularly among those derived for workers, which likely do not provide adequate protection for asthmatics. Copyright © 2016 The Authors *Journal of Applied Toxicology* Published by John Wiley & Sons Ltd.

## Abstract 2

We investigate the extent to which experimental observations on asthmatic subjects are taken into consideration by REACH registrants and determine whether asthmatics are provided adequate protection by the acute inhalation Derived No‐Effect Levels. Of 114 studies concerning 14 substances fulfilling our inclusion criteria, 63 studies, involving nine substances, were cited by the REACH registrants. However, only 17 of the 114 studies, involving four substances, were regarded as key studies. Furthermore, many of the Derived No‐Effect Levels were higher than Lowest‐ or No‐Observed Adverse Effect Concentrations values from these studies.

## Introduction

The European regulation REACH (EC, 1907/2006) concerning the registration, evaluation, authorization and restriction of chemicals entails major change in the approach to the regulation of industrial chemicals. As stated in article 1(3) of the regulation it “is based on the principle that it is for manufacturers, importers and downstream users to ensure that they manufacture, place on the market or use such substances that do not adversely affect human health or the environment.” This responsibility includes ensuring the availability of relevant toxicological data, as well as evaluation of these data in attempt to establish guidelines for safe use.

A key part of this evaluation is the determination of derived no‐effect levels (DNELs) (i.e,, the levels of exposure below which no adverse health effects in humans are expected to occur). Registrant companies are required to provide DNELs for all substances they manufacture and/or import in quantities of more than 10 tons per year. To date, two of the three deadlines for registration have expired (the third being in 2018) and the extensive effort expended by registrants is illustrated by the more than 51 000 dossiers covering more than 13 000 unique substances that were submitted by June 2015. Via the database of registered substances, the European Chemicals Agency (ECHA website, 2016) disseminates selected information from these dossiers (ECHA, 2012a), including the DNELs themselves and summaries of the toxicological data on which these are based.

In chapter R.8 (ECHA, 2012b) the comprehensive guidance developed for REACH obligation‐holders, the procedure by which DNELs should be derived is outlined. These values should consider relevant populations (workers, consumers or other individuals liable to be exposed indirectly via the environment, as well as certain susceptible and/or vulnerable groups, such as pregnant women and children), the type of toxic effects (local or systemic), routes of exposure (inhalation, oral and/or dermal) and duration of exposure (long‐ or short‐term/acute). Health‐based occupational exposure limits (OELs) developed previously, for example, those recommended by the Scientific Committee on Occupational Exposure Limits (SCOEL) of the European Commission, may under certain circumstances be utilized as inhalation DNELs for workers, as long as these are not put into question by new scientific findings (ECHA, 2012b, p.137).

The duration of exposure taken into consideration should be relevant to the manner in which the substance is used. The present investigation focused on pulmonary effects caused by acute inhalation of chemicals. DNELs for acute inhalation are generally based on minutes to a few hours of exposure, with the ECHA guidance specifying a routine duration of 15 min for workers (ECHA, 2012b, p.102–104). In the case of consumers, the ECHA guidance defines a daily dose as that resulting from 1 to 24 h of exposure, with peak levels of exposures lasting minutes to hours (ECHA, 2012b, p.8).

In connection with the derivation of DNELs, the ECHA guidance contains default assessment factors (AFs) designed to adjust for certain aspects of uncertainty and variability. The default intraspecies AF for workers is set to 5, in contrast to the value of 10 for the general population. The factors applied may deviate from the default values. Thus, inclusion of a susceptible subpopulation may necessitate a higher AF than the default value. Conversely, a reduced intraspecies AF may be appropriate if the point of departure is based on a study where appropriate susceptible groups (e.g., asthmatics) are already included (ECHA, 2012b, p.160).

It is important to take asthmatics into consideration in connection with risk assessment of inhalation exposure, as they constitute a significant portion of the population and, moreover, are susceptible to certain health effects. The World Health Organization (WHO) reports that approximately 235 million people worldwide have asthma, making this currently one of the most common chronic diseases (WHO, 2015 2013). Variations in diagnosis make it difficult to determine the prevalence exactly. One survey of available studies arrived at a 1–18% prevalence of clinical asthma globally, with a prevalence of more than 5% in many countries of western Europe (Masoli *et al*., 2004). Although the prevalence of asthma among workers may be similar to that among the general population (or even somewhat lower, due to the hindrance that asthma presents to work), few studies have yet addressed this question. Among 474 workers in Maine, 13.5% currently had asthma (either as diagnosed by a physician or based on reported symptoms characteristic of this disease (Henneberger *et al*., 2003), a value relatively similar to the 10.9% reported by Masoli and colleagues for the entire population of the United States.

In addition to allergens, a variety of factors, including irritant chemicals, dusts and second‐hand smoke, can exacerbate pre‐existing asthma (Papaioannou *et al*., 2015; WHO, 2015 2013). Accordingly, asthmatics may suffer adverse effects at lower levels of exposure to airborne chemicals than healthy individuals. Indeed, such enhanced susceptibility has been demonstrated in several studies, including our recent analysis of differences in the responses of healthy and asthmatic airways during acute exposure to airborne chemicals (Johansson *et al*., 2016). We have previously concluded that available experimental findings indicate that an intraspecies AF of 10 (e.g., as proposed by ECHA for the general population but not for workers) is adequate for protection of asthmatic individuals (Johansson *et al*., 2016).

Specific work environments can contribute to the development of asthma and/or exacerbation of this disease. The American Thoracic Society (ATS) states that “…there may be much greater morbidity and productivity loss associated with exacerbations of pre‐existing asthma due to workplace exposures than due to de novo asthma caused by such exposure…” (ATS, 2003). In this context, Henneberger et al. (2015) have shown that asthma in working adults can be severely exacerbated by several occupational agents, in particular irritants. In light of the pronounced incidence of asthma and this potential impact of the working environment, asthmatics should be taken into consideration in connection with derivation of DNELs for respiratory irritants.

None the less, despite such observations, as well as the considerable socio‐economic burden posed by this disease (Bahadori *et al*., 2009), exposure data concerning asthmatics are frequently disregarded in connection with the derivation of acute guidelines, for either the general and/or working population (Johansson *et al*., 2012). Different programmes for risk assessment target different populations and have different policies with respect to the inclusion of susceptible groups such as asthmatics. As shown in Table 1, most such programmes targeting the general population appear to include susceptible (although not hypersusceptible) groups and a few mention asthmatics specifically.

**Table 1:** Target groups taken into consideration with the derivation of acute to short‐term guideline values for the general and working populations

Guideline value	Organization	Target population
Derived no‐effect levels (DNEL)	REACH registrants	“…it may be necessary to identify different DNELs for each relevant human population (e.g., workers, consumers and humans liable to exposure indirectly via the environment) and possibly for certain vulnerable sub‐populations (e.g., children, pregnant women)” (ECHA, 2012b, p.2)
**Acute to short‐term values for the general population**		
Acute exposure guideline levels (AEGL)	US National Research Council and Environmental Protection Agency (NRC/EPA)	“…general public, including susceptible subpopulations, such as infants, children, the elderly, persons with asthma, and those with other illnesses…” (NRC, 2001, p.3)
Emergency response planning guidelines (ERPG)	American Industrial Hygiene Association (AIHA)	“… should not be expected to protect everyone, but should be applicable to most individuals in the general population. In all populations, there are hypersensitive individuals who will show adverse responses at exposure concentrations far below levels at which most individuals normally would respond.“ (AIHA, 2008, p.14)
Minimal risk levels (MRL)	US Agency for Toxic Substances and Disease Registry (ATSDR)	“Conditions that may enhance susceptibility to adverse health effects include age, sex, genetic composition, nutritional status, and pre‐existing disease conditions. UFs of 10 are usually used to derive MRLs protective of these sensitive subpopulations.” (Pohl & Abadin, 1995)
Reference exposure levels (REL)	California Office of Environmental Health Hazard Assessment (OEHHA)	“both individuals at low risk for chemical injury as well as identifiable sensitive subpopulations (highly susceptible or sensitive individuals)… […]. .asthmatics are frequently identified as a sensitive group.” (Denton & Hickox, 1999, p.13)
French acute toxicity threshold values (VSTAF)	French National Institute for Industrial Environment and Risks (INERIS)	“… general population excluding susceptible and hypersusceptible individuals” (INERIS, 2009, p.14)
**Acute to short‐term values for workers**		
Dutch Expert Committee on Occupational Standards (DECOS)	Health Council of the Netherlands	“…health‐based recommended occupational exposure limit for the concentration of the substance in workroom air.” (DECOS, 2000, p.4) No further details are included.
Maximum concentration at the workplace (MAK)	German Research Foundation (DFG)	“The diverse sensitivities of individual employees (as determined by age, constitution, nutrition, climate, etc.) are taken into consideration in the establishment of MAK values.” (DFG, 2014, p.14)
Scientific Committee on Occupational Exposure Limits (SCOEL)	European Commission	“Groups at higher risk in relation to a specific compound will be identified in the corresponding recommendation and available information provided, but the OELs are established for healthy workers.” (SCOEL, 2013, p.16)
Swedish occupational exposure limits (SE‐OEL)	Swedish Work Environment Authority (SWEA)	Workers. No further details are included in AFS (2011)
Threshold limit values (TLV)	American Conference of Governmental Industrial Hygienists (ACGIH)	“…workers who are normal, healthy adults.” (ACGIH, 2015)

For example, the acute exposure guideline level (AEGL) standing operating procedures specify clearly that the potentially elevated sensitivity of asthmatic individuals should be taken into consideration when deriving guidelines (NRC, 2001). However, in a previous study we have shown that 123 of 176 technical support documents for substances with AEGL values lack any explicit statement concerning these individuals (Johansson *et al*., 2012).

The occupational short‐term values and limits presented in Table 1 have been developed for the working population and no information concerning asthmatics was included in any of the reviewed methodologies of these values (ACGIH, 2015; AFS, 2011; DECOS, 2000; DFG, 2014; SCOEL, 2013). However, the DFG states that variations in the sensitivity of individual employees should be considered. On the other hand, the SCOEL and ACGIH state specifically that their values concern healthy workers, i.e., asthmatics are presumably excluded. As SCOEL OEL recommendations are accepted as a potential alternative to DNELs, registrants may assume that asthmatics need not be taken into consideration when deriving worker DNELs either.

As REACH is a relatively new attempt to regulate safe use of chemicals, it is of considerable interest to examine how asthmatics are considered by REACH registrants, particularly in comparison to programmes for deriving guidelines for acute or short‐term exposure. Here, we evaluate the extent to which experimental data on asthmatics have been included in REACH registrations and utilized to derive DNELs for acute inhalation exposure. Furthermore, we estimate no observed adverse effect concentrations (NOAEC) and lowest observed adverse effect concentrations (LOAEC) for various substances for asthmatic individuals based on all available studies. These overall NOAEC and LOAEC values have then been compared with the DNELs and other guideline values for acute exposure to ascertain whether asthmatics are adequately protected at present.

## Methods

### Selection of substances

The starting point for the present study was the availability of published experimental data on asthmatics under controlled exposures. Substances were selected based on our previous search for studies comparing healthy and asthmatic human subjects (in the technical support documents from 10 risk assessment programmes and in PubMed, Toxnet, EMBASE and Google Scholar; Johansson *et al*., 2012, 2016), as well as further expansion here to also include nine additional studies with asthmatics only (latest search performed in April 2015). In addition, we examined all 269 substances with classification H335 – respiratory irritant under the Classification, Labelling and Packaging Regulation (C&L Inventory, website).

In total, we were able to identify 57 substances with experimental data from controlled exposures including asthmatic subjects. These 57 cover 22 ‘primary substances’, i.e., on which experimental data have been published, and 35 ‘secondary substances’, whose reaction products are among the primary 22 or which are structural analogues of these primary substances. Finally, among the 57, we identified 23 substances, 14 primary and nine secondary, in the ECHA database of Registered substances (as of April 2015).

### Compilation of experimental studies, including asthmatic subjects

Experimental studies including asthmatic subjects were searched for in the Web of Knowledge, PubMed and Google Scholar. Employing the name of one of the 23 chemicals and the terms: ‘asthma’, ‘exposure’, ‘NOT mice’ and ‘NOT rat’. Only human studies on asthmatics involving controlled exposure to a single substance were assessed.

Epidemiological studies on air pollution and studies in occupational settings, where exposure is not adequately defined, were excluded. Investigations in which subjects with occupational asthma were exposed to the sensitizing chemical were also excluded, as were duplicates and conference abstracts. The subjects were diagnosed with asthma by a physician in most of the original experimental papers. In some cases, the diagnosis was based on a medical history of asthma. Exposures were performed either during exercise or at rest and either breathing freely or via mouth‐only (wearing nose‐clip). Most studies included subjects in the age interval 18–56. Additional details are given as Supplemental material.

The final search performed on 29 April 2015, yielded 153 original papers. Consideration of the design and technical limitations of each study revealed that 114 studies (listed in Supplemental material) fulfilled the following criteria: publication in a peer‐reviewed journal; experiment carried out at normal indoor humidity and at normal room temperature (18–25 °C). Exclusions were: current smokers; those diagnosed with severe asthma (according to authors of original study); studies where sensitized asthmatic individuals were exposed to the sensitizer; subjects with diseases other than asthma (e.g., chronic obstructive pulmonary disease); and anyone with known previous extensive exposure to irritating or sensitizing air pollutants (e.g., at the work place).

From each of these 114 studies, we extracted NOAECs and LOAECs for pulmonary function and/or respiratory symptoms. Moreover, based on all relevant information available, overall NOAECs and LOAECs were derived for each chemical taking the number of subjects, duration and conditions of exposure and endpoints monitored into consideration. More weight was given to studies involving more subjects and very short exposures were considered inconclusive. Effects induced by exercise were only included if controlled for by comparison to responses to pure, filtered air. In addition, parameters of pulmonary function, such as the forced expiratory volume in 1 s (FEV_1_) and specific airway resistance, were considered more relevant than self‐reported irritation. As a large number of investigations of reliable quality were available for sulphur dioxide and sulphuric acid the medians of all their FEV_1_ values were identified as overall NOAECs and LOAECs (Supplemental Material; see Table S1).

### Compilation of derived no‐effect levels and related information

DNEL values and publicly available information concerning their derivation (the AFs applied, references to all experimental reports), as well as the harmonized and notified classifications listed in the C&L Inventory were collected for each of the 23 substances from the publicly available ECHA database of registered substances (ECHA website). For comparison, priority was assigned to DNELs for local effects from acute/short‐term inhalation exposure, as such effects are observed in asthmatics (Supplemental Material; see Table S1). For three of the registered substances, no DNEL for local effects following acute inhalation exposure was available in April 2015, but DNELs for other effects and durations of exposure were available (as pointed out in Table 4). DNELs for workers and general populations were compiled to enable comparisons to other acute values. For each of the 23 substances the toxicological information reported in the ECHA database of registered substances was searched utilizing the term ‘asthma’ to identify studies involving asthmatics taken into consideration by the registrant (available at ECHA website, search performed on 21 April 2015).

The asthma studies found in the ECHA database of registered substances were compared to our compilation of studies (Supplemental Material; see Table S1). Then, the DNEL values for local effects following acute/short‐term exposure of both workers and the general population were compared to the 10 health‐based guidelines described previously (Johansson *et al*., 2012). In addition, the DNEL values were compared to overall NOAECs and overall LOAECs estimated for each chemical here.

## Results

Experimental data on asthmatic subjects were available for 23 substances registered under REACH (Table 2). We found 114 original experimental investigations related to these chemicals involving measurements of airway irritation and pulmonary function in asthmatic subjects.

**Table 2:** Hazard information on registered substances for which experimental data concerning asthmatics are available

Substance		Respiratory irritant according to HSDB^a^	Notified classification H335 (resp. tract) in the C&L inventory	Harmonised classification H335 (resp. tract) in the C&L inventory	Harmonised classification of health hazards in the C&L inventory database^c^
Name	CAS no.				
Acetaldehyde	75–07‐0	Yes	Yes	Yes	Eye Irrit. 2 H319, STOT SE 3 H335, Carc. 2 H351
Ammonia	7664–41‐7	Yes	Yes	No	Skin Corr. 1B H314, Acute Tox. 3 *H331
Ammonium sulphate	7783–20‐2	Yes	Yes	–	–
Chlorine	7782–50‐5	Yes	Yes	Yes	Skin Irrit. 2 H315, Eye Irrit. 2 H319, Acute Tox. 3*H331 , STOT SE 3 H335
Bromine (structure analogy: chlorine)	7726–95‐6	Yes	Yes	No	Skin Corr. 1 A H314, Acute Tox. 2 * H330
Diiron tris(sulphate) (ferric sulphate)	10028–22‐5	Yes	Yes	–	–
Formaldehyde	50–00‐0	Yes	Yes	No	Acute Tox. 3 * H301, Acute Tox. 3 * H311, Skin Corr. 1B H314, Skin Sens. 1 H317, Acute Tox. 3 * H331, Muta. 2 H341, Carc. 1B H350
Hydrogen chloride	7647–01‐0	Yes	Yes	Yes	Press gas: Skin Corr. 1 A H314, Acute Tox. 3 * H331Hydrochloric acid: Skin Corr. 1B H314, STOT SE 3 H335
Hydrogen bromide (structure analogy: hydrogen chloride)	10035–10‐6	Yes	Yes	Yes	Skin Corr. 1 A H314, STOT SE 3 H335
Octyl trichlorosilane (forms hydrogen chloride upon hydrolysis)	5283–66‐9	Yes	Yes	–	–
Silicon tetrachloride (forms hydrogen chloride upon hydrolysis)	10026–04‐7	Yes	Yes	Yes	Skin Irrit. 2 H315, Eye Irrit. 2 H319,STOT SE 3 H335
Trichlorosilane (forms hydrogen chloride upon hydrolysis)	10025–78‐2	Yes	Yes	No	Acute Tox. 4 * H302, Skin Corr. 1 A H314, Acute Tox. 4 * H332
Hydrogen sulphide	7783–06‐4	Yes	Yes	No	Acute Tox. 2 * H330
Nitric acid	7697–37‐2	Yes	No	No	Skin Corr. 1 A H314
Dinitrogen tetroxide (nitrogen tetroxide) (vaporizes and dissociates into nitrogen dioxide in air.)	10544–72‐6	Yes	Yes	No	Skin Corr. 1B H314, Acute Tox. 2 * H330
Sodium hydrogen sulphate (sodium bisulphate)	681–38‐1	– b	No	No	Eye Dam. 1 H318
Sodium nitrate	7631–99‐4	Yes	Yes	–	–
Sulphur dioxide	7446–09‐5	Yes	Yes	No	Skin Corr. 1B H314, Acute Tox. 3 * H331
Thionyl chloride (structure analogy: sulphur dioxide)	7719–09‐7	Yes	Yes	No	Acute Tox. 4 * H302, Skin Corr. 1 A H314, Acute Tox. 4 * H332
Sulphuric acid	7664–93‐9	Yes	Yes	No	Skin Corr. 1 A H314
Sulphur trioxide (forms sulphuric acid upon hydrolysis)	7446–11‐9	Yes	Yes	–	–
Chlorosulphonic acid (structure analogy: sulphuric acid)	7790–94‐5	Yes	Yes	Yes	Skin Corr. 1 A H314, STOT SE 3 H335
4‐Methyl‐m‐phenylene diisocyanate (2,4‐toluene diisocyanate)	584–84‐9	Yes	Yes	Yes	Skin Irrit. 2 H315, Skin Sens. 1 H317, Eye Irrit. 2 H319, Acute Tox. 2 * H330, Resp. Sens. 1 H334, STOT SE 3 H335 Carc. 2 H351

–, classification not available; Carc, carcinogenicity; Corr, corrosion; Dam, damage; Irr , irritation; Muta, mutagenicity; Press gas, gas under pressure; Resp, respiratory; Sens, sensitization; Tox, toxicity; STOT SE, specific organ toxicity single exposure.

a Hazardous Substances Data Bank, HSDB website search performed 9 September 2015.

b Not available in HSDB. Classified as a respiratory irritant by the European Food Safety Authority (EFSA) (2011).

c C&L Inventory website. Search performed 17 June 2015. Harmonised classifications are also included in Annex VI to the Regulation on Classification, Labelling and Packaging. The regulation also includes definitions of hazard phrases listed in this table and classification criteria. An unofficial consolidated version of the regulation is available at: http://eur‐lex.europa.eu/legal‐content/EN/TXT/?uri=CELEX:02008R1272‐20150601 (accessed 23 March 2016).

Only seven are considered respiratory irritants (H335) according to the harmonized classification (Table 2). However, 22 are identified as respiratory irritants (H335) by one or more registrants. It should be noted that for the remaining substance, 2,4‐toluene diisocyanate (registered as 4‐methyl‐m‐phenylene diisocyanate), classified as a respiratory sensitizer (H334, R42/43), the only study on exposure available involved non‐sensitized subjects (Table 2; Supplemental Material, see Table S1) At the same time, all 23 substances are identified as respiratory irritants in the hazardous substances data base (HSDB), by the US National Library of Medicine (HSDB, website) and/or by the European Food Safety Authority (EFSA) Panel on Additives and Products or Substances used in Animal Feed (EFSA, 2011).

Of the 114 experimental investigations analysed here, only 63 covering nine of the 23 substances are cited by the REACH registrants (Table 3). Most of the available studies concerned sulphur dioxide, and, accordingly, risk assessments for this substance generally include the largest number of different studies on asthmatic subjects (Table 3). Among the nine substances was ferric sulphate [registered as diiron tris(sulphate)], for which no DNEL values for local effects following acute inhalation exposure were available (Table 4).

**Table 3:** Inclusion of relevant experimental data on asthmatic subjects by REACH registrants and by 10 other groups of risk assessment experts

Substance	Number of experimental studies on asthmatics					
	Found in the literature	Cited in risk assessment documents from 10 sets of acute or short‐term values^a^	Used as key studies in risk assessment documents from 10 sets of acute or short‐term values^a^	Cited by registrant at the ECHA website	Used as key study by registrant	Reliability of study according to registrant^b^
Acetaldehyde	11	9 (AEGL, ERPG, REL)	1 (REL)	–	–	–
Ammonia	2	2 (AEGL, REL, SE‐OEL)	1 (AEGL)	–	–	–
Ammonium sulphate	7	No document	No document	2	2	2, 2
Chlorine	1	1 (AEGL, ERPG, MRL, REL, SCOEL)	1 (AEGL, ERPG, MRL, REL, SCOEL)	–	–	–
Bromine (structure analogy: chlorine)	1	1 (AEGL)	1 (AEGL)	–	–	–
Diiron tris(sulphate)	1	1 (DECOS)	1 (DECOS)	1	–	2
Formaldehyde	12	11 (AEGL, ERPG, MRL, REL, VSTAF, DECOS, MAK, SE‐OEL, TLV)	4 (AEGL)	6	–	2, 2, 2, 2, 2, 2
Hydrogen chloride	1	2 (AEGL, ERPG, REL, VSTAF, SE‐OEL, TLV)	1 (AEGL, ERPG, REL, TLV)	–	–	–
Hydrogen bromide (structure analogy: hydrogen chloride)	1	1 (AEGL)	–	–	–	–
Octyl trichlorosilane (forms hydrogen chloride upon hydrolysis)	1	1 (AEGL)	1 (AEGL)	–	–	–
Silicon tetrachloride (forms hydrogen chloride upon hydrolysis)	1	1 (AEGL)	1 (AEGL)	–	–	–
Trichlorosilane (forms hydrogen chloride upon hydrolysis)	1	1 (AEGL)	1 (AEGL)	–	–	–
Hydrogen sulphide	1	1 (AEGL, MRL, REL, VSTAF, DECOS, SCOEL)	1 (AEGL, MRL)	1	–	4
Nitric acid	4	5 (AEGL, ERPG, REL, VSTAF, SE‐OEL)	2 (ERPG, REL)	1	–	4
Dinitrogen tetroxide (vaporizes and dissociates into nitrogen dioxide in air)	44	1 (AEGL)	1 (AEGL)	–	–	–
Sodium hydrogen sulphate	3	No document	No document	–	–	–
Sodium nitrate	3	No document	No document	–	–	–
Sulphur dioxide	66	48 (AEGL, ERPG, MRL, REL, VSTAF, DECOS, SCOEL, SE‐OEL)	10 (AEGL, ERPG, MRL, REL, DECOS, SCOEL)	46	11	1 (35), 2 (11)
Thionyl chloride (structure analogy: sulphur dioxide)	66	2 (AEGL)	–	2	2	Not stated
Sulphuric acid	38	38 (AEGL, ERPG, REL, VSTAF, MAK, SCOEL, SE‐OEL, TLV)	7 (AEGL, ERPG, REL, TLV)	2	–	2, 2
Sulphur trioxide (forms sulphuric acid upon hydrolysis)	38	28 (AEGL)	2 (AEGL)	2	2	2, 2
Chlorosulphonic acid (structure analogy: sulphuric acid)	38	28 (AEGL)	2 (AEGL)	–	–	–
4‐methyl‐m‐phenylene diisocyanate	2	2 (AEGL, ERPG, MAK, SE‐OEL)	1 (AEGL, ERPG)	–	–	–

–, not cited; AEGL, acute exposure guideline levels; DECOS, limits recommended by the Dutch Expert Committee on Occupational Standards; ERPG, emergency response planning guidelines; MAK, maximum concentration at the workplace; MRL, minimal risk levels; REL, reference exposure levels; SCOEL, limits recommended by the Scientific Committee on Occupational Exposure Limits; SE‐OEL, Swedish occupational exposure limits; TLV, threshold limit values; VSTAF, French acute toxicity threshold values.

a As reported in Johansson *et al*. (2012).

b Given as Klimisch codes: 1 = reliable without restrictions; 2 = reliable with restrictions; 3 = not reliable; 4 = not assignable (Klimisch *et al*. 1997).

**Table 4:** Comparison of DNEL values with 10 short‐term values for workers and the general population (mg m^−3^). LOAEC and NOAEC values are derived from our compilation of experimental studies on asthmatics (Appendix A Table A1). Inhalation DNEL values are for local effects from acute/short‐term exposure, if not otherwise specified

	Based on asthma data		Acute/short‐term values for the general population						Acute/short‐term values for workers					
Substance	Estimated LOAEC	Estimated NOAEC	DNEL(Feb 25 2015)	AEGL10, 30, 60, 120, 240, 480 min(May 2015)	ERPG11 h(2013)	MRL1–14 d(Dec 2014)	REL1 h(Jun 2014)	VSTAF1, 3, 10, 20, 30, 60 min(2015)	DNEL(Feb 25 2015)	DECOS 15 min (2013)	MAK 15 min(Aug 2014)	SCOEL 15 min(May 2013)	SE‐OEL15 min(2011)	TLV15 min(2010)
Acetaldehyde	–	–	NA	81	18	–	0.47	–	NA	–	91	–	90	45^c^
Ammonia	–	11.3–14.2	7.2	21	17.5	1.2	3.2	196, 140, 105, 84, 77, 56	36	–	28	36	36^c^	24
Ammonium sulphate	1	0.5	1.7^a^	–	–	–	–	–	11.2^a^	–	–	–	–	–
Chlorine	2.9	1.16	1.5	1.5	2.89	0.17	0.21	–	1.5	–	1.5	1.5	3^c^	2.9
Bromine	–	–	NA	0.22	0.7	–	–	NA	0.7	0.2	–	–	2	1.3
Diiron tris(sulphate)	–	0.075	NA	–	–	–	–	–	NA	NA	–	–	–	–
Formaldehyde	3.7	2.48	0.1^b^	1.1	1.23	0.049	0.055	1.25–2.5	1	3	0.74	0.49	0.74^c^	0.37^c^
Hydrogen chloride	–	2.68	NA	2.7	4.5	–	2.1	NA	15	–	6.0	15	8^c^	2.98^c^
Hydrogen bromide	–	–	NA	3.3	–	–	–	NA	6.7	–	6.7	6.7	7^c^	6.6^c^
Octyl trichlorosilane	–	–	NA	0.9	–	–	–	–	18	–	–	–	–	–
Silicon tetrachloride	–	–	NA	0.68	5.3	–	–	–	9.3^b^	–	–	–	–	–
Trichlorosilane	–	–	NA	0.9	5.5	–	–	–	0.23	–	–	–	–	–
Hydrogen sulphide	–	2.8	NA	1.05, 0.84, 0.71, 0.5, 0.46	0.14	0.098	0.042	NA	14	8 h: 2.3	14.2	14	20^c^	7
Nitric acid	0.13	–	1.3	1.4	3	–	0.086	–	2.6	–	5	2.6	13	10
Dinitrogen tetroxide	–	–	NA	0.94	–	–	–	–	0.17^a^	–	–	–	–	–
Sodium hydrogensulphate	1	0.45	NA	–	–	–	–	–	NA	–	–	–	–	–
Sodium nitrate	–	7	10.9^a^	–	–	–	–	–	36.7^a^	–	–	–	–	–
Sulphur dioxide	Median 2.15	Median1.4	0.53^b^	0.52	0.78	0.026	0.66	7.8	2.7	0.7	2.7	2.7	13^c^	0.65
Thionyl chloride	–	–	NA	NA	0.97	–	–	NA	1	NA	–	–	–	1^c^
Sulphuric acid	Median: 0.45	Median: 0.21	NA	0.2	2	–	0.12	–	0.1	–	0.1	(0.1)	0.2	8 h:0.2
Sulphur trioxide	–	–	NA	0.2	2	–	–	–	0.1	–	–	–	–	–
Chlorosulphonic acid	–	–	0.01^a^	0.1	2	–	–	–	0.04^a^	–	–	–	–	–
4‐methyl‐m‐phenylene diisocyanate	–	0.142^d^	NA	10–60 min: 0.14, 240–480 min: 0.07	0.07	–	–	1, 10 min: 7.1 20–60 min: 0.71	0.14^d^	–	0.02	–	0.04^c^	0.14

–, no LOAEC or no risk assessment available; AEGL, acute exposure guideline levels; DECOS, limits recommended by the Dutch Expert Committee on Occupational Standards; DNEL, derived no‐effect levels; ERPG, emergency response planning guidelines; LOAEC, lowest observed adverse effect concentrations; MAK, maximum concentration at the workplace; MRL, minimal risk levels; NA, not assigned (risk assessment document available but no values proposed); NOAEC, no‐observed adverse effect concentrations; REL, reference exposure levels; SCOEL, limits recommended by the Scientific Committee on Occupational Exposure Limits; SE‐OEL, Swedish occupational exposure limits; TLV, threshold limit values; VSTAF, French acute toxicity threshold values.

a Systemic effects of long‐term exposure.

b Local effects of long‐term exposure.

c Ceiling limit.

d For respiratory sensitizers, the guidance document states that DNELs are not applicable, as thresholds cannot be established. Therefore, the DNEL values for 4‐methyl‐m‐phenylene diisocyanate, which is classified as respiratory sensitizer, appear to be erroneous.

Data on asthmatics were flagged as key studies by the REACH registrants for only four substances (Table 3). However, the issues of data coverage and key study selection are further complicated by the fact that DNELs may be derived from SCOEL short‐term exposure limit values and, indeed, for eight substances these two values were identical (Table 4). Where the acute/short‐term DNEL for workers was identical to a short‐term OEL, the SCOEL document on that substance was examined in greater detail, since, as previously mentioned, in certain cases an OEL may be used as a surrogate worker DNEL (ECHA, 2012b, p.137).

For substances chlorine, hydrogen sulphide, sulphur dioxide and sulphuric acid, SCOEL did take asthmatics into consideration and, in fact, key consideration in two cases (chlorine and sulphur dioxide). However, the ECHA database of registered substances did not contain any data on asthmatics exposed to chlorine. Assuming that the DNEL for chlorine is actually taken from the SCOEL short‐term exposure limit, evaluations, including asthmatics, were employed to derive DNEL values for a total of 10 substances (Tables 3 and 4).

In the development of acute exposure guidance/limit values by other risk assessment programmes (Table 4), data on asthmatics from one or more toxicological evaluations are cited for 20 of the 23 substances and used as key data at least once for 18 of these 20 (Table 3). Three of the 23 substances, i.e., ammonium sulphate, sodium bisulphate and sodium nitrate, have not yet been evaluated in any of these other programmes.

In accordance with its explicit aim in this context, AEGL is the risk assessment programme that relies most extensively on information concerning asthmatics. Such studies are cited in all the 19 AEGL risk assessments analysed here and stated to be key data in 15 of these. The California Office of Environmental Health Hazard Assessment reference exposure levels also takes studies on asthmatics into consideration relatively often, i.e., all of the documents included here, and as key data in six of these. Other risk assessors have included investigations on asthmatics to a lesser extent (Table 3).

Inhalation DNELs for workers and/or the general population are available for 20 substances and missing for the three others (Table 4). Overall, these DNEL values do not differ distinctly from the other 10 sets of acute/short‐term guideline values. In addition, the SCOEL short‐term values and DNELs for the worker population are in all cases, excluding formaldehyde, identical (Table 4).

We were able to estimate overall LOAECs for asthmatics for six of the 20 substances that had an inhalation DNEL. For chlorine, sulphur dioxide and sulphuric acid the overall LOAECs correspond to moderate responses according to the US EPA (2007, 2008, 2009), i.e., a reduction in FEV_1_ between −10 and −20% and/or an increase in specific airways resistance between 100 and 200% (e.g., D'Alessandro *et al*., 1996; Linn *et al*., 1977; Utell *et al*., 1989). For nitric acid, the overall LOAEC was derived from a study where lung function measurements showed relatively mild effects, e.g., a reduction in FEV_1_ of 4.4% (Koenig *et al*., 1989). For ammonium sulphate, only one study reported of statistically significant changes in lung function, unfortunately not reported as changes in FEV_1_, the decrease in flow rates was described by study authors as ‘quite small’ and airway obstruction ‘definitive but asymptomatic’ (Utell *et al*., 1982). The overall LOAEC for formaldehyde is derived primarily from symptoms of irritation seen in Sauder *et al*. (1987).

For two of the substances with an overall LOAEC (ammonium sulphate and nitric acid), DNELs for both workers and the general population are higher than the overall LOAEC. For a third substance (sulphur dioxide) the worker DNEL is higher than the overall LOAEC. In the case of nitric acid, all acute guideline values investigated except REL exceed the overall LOAEC by at least a factor of 10. For sulphur dioxide, three of five occupational short‐term limits exceed the overall LOAEC (Table 4). Regarding those six substances with an overall NOAEC only (and no LOAEC), the DNEL is higher in four cases (including ammonia, for which the DNEL for the general population is lower but the worker DNEL is higher than the overall NOAEC). Guideline values for workers more often exceed the overall NOAECs than do the corresponding values for the general population (Table 4).

## Discussion

We examined whether asthmatics are taken into consideration in connection with the derivation of DNELs for both the general and working populations for local effects of acute/short‐term exposure to chemicals through inhalation.

A major current observation is that experimental data on asthmatic individuals is generally lacking, as we also noted earlier (Johansson *et al*., 2012). Of the 269 substances identified as respiratory irritants by the harmonized classification of the Classification, Labelling and Packaging Regulation, only 2.6% were found to have been tested on asthmatic subjects. The ECHA database of registered substances includes only 14 of the 22 substances that had been tested directly on asthmatic subjects, in addition to nine reaction products or structural analogues. This lack of experimental data constitutes a limitation of risk assessments for airborne chemicals in general and of the present study.

Experimental findings on asthmatics are either key studies or, more often, supporting data, for 10 of the 23 substances in the ECHA database of registered substances. Thus, in many of our cases relevant data on asthmatics appear to have been disregarded by the registrants. In this context, the REACH registrants resemble the risk assessments programmes that derive acute to short‐term values for workers more closely than those that derive guideline values for the general population were. It should also be noted that among the 114 studies reviewed here the most recent was published in 2007 (Supplemental Material, see Table S1), so the REACH registrants could have accessed all of these articles before the first registration deadline in 2010.

The DNELs investigated here, were neither systematically higher nor lower than the corresponding guideline values for acute/short‐term exposure recommended by the other 10 risk assessment programmes, and, indeed, there was a striking similarity to the SCOEL for worker DNELs (Table 4). These observations are analogous to those of previous reports that long‐term inhalation DNELs for workers may be either much lower or much higher than the corresponding national 8 h OELs, but are generally similar to the corresponding SCOEL values (Nies *et al*., 2013; Schenk *et al*., 2015).

Overall, guidelines for workers, including the DNELs here, are generally higher than those for the general population were. As demonstrated previously, discrepancies in guidelines may not only arise from different target populations and availability of data, but also from differing criteria for data selection and evaluation, methodology and risk assessing experience (Öberg *et al*., 2010; Schenk, 2010). Each substance has its own registrants and it is not surprising that interpretation and application of the ECHA guidance differ between registrants. Given the fact that only 5% of the dossiers are scheduled to be examined by the ECHA, it is important to establish clear guidance documents that minimize arbitrariness.

In addition, we note that the difference between DNELs for the general population and those for workers are often larger than the factor 2, which would be the outcome when applying the recommended default AFs of 10 for the general population and five for workers (Table 4). Contrary to this observation, it may even be the case that for exacerbation of asthma different AFs for the general population and workers are not warranted (i.e., a factor of 10 should be applied also for workers, cf. Johansson *et al*., 2016). In the present work, we have assumed that the estimates of LOAECs and NOAECs are relevant for mild asthmatics within the general as well as the working population.

In our evaluation of whether asthmatics are likely to be protected by the DNELs reviewed (Table 4), we found inhalation values for 20 substances. For three substances, the worker DNELs were higher than our estimated overall LOAECs; for two of these also the DNELs for the general population were higher than the overall LOAEC. The overall LOAECs derived in the present paper should not be seen as alternative exposure guidelines. Rather, we argue that exceeding an overall LOAEC established at levels of moderate to severe airway response indicates that the exposure guidelines are not sufficient for the protection of asthmatics. Differences of more than a factor of 10 between overall LOAEC and DNEL, were found for nitric acid for both the general population and workers as well as for ammonium sulphate for workers (Table 4). Hence, even though the overall LOAECs for these two substances are derived from comparatively mild airway responses (Koenig *et al*., 1989; Utell *et al*., 1982) we conclude that these DNELs offer a dubious level of protection for asthmatics.

For eight substances, the DNEL for the general population and/or workers was set close to or higher than the estimated overall NOAEC, indicating small or no safety margins. However, it is difficult to draw any firm conclusions regarding safety margins, as many of the controlled studies in our compilation involved few subjects and exposure to a single concentration only. Thus, for six of these eight substances it was not possible to derive an overall LOAEC (Supplemental Material, see Table S1).

Absence of or very low safety margins may lead to asthmatics experiencing exacerbations during a regular workday or even in common situations as consumers. DNELs are not meant for rare exposures, but rather exposures repeated during a working‐life or lifetime. One question is whether such low safety margins are acceptable in connection with such long time frames. Moreover, the subjects in the 114 studies reviewed here were considered to have mild asthma, so the LOAEC and NOAEC values may be inappropriate for individuals with severe asthma. However, it should be noted that the safety margin at the use stage depends also on the operationalization of the DNEL, i.e., defining allowed uses and prescribed risk management measures for the substances in question.

Our present evaluation raises several questions concerning how standards are set, and not only in connection with REACH registration. For example, which regulatory frameworks (e.g., REACH, occupational health regulations, product‐specific regulations) should take asthmatics explicitly into consideration, and, in what way should this be done?

The aims listed in Article 1 of REACH do not mention susceptible subpopulations (EC, 1907/2006). Whether, and to what degree, asthmatics should be protected by DNELs are important questions for policy makers. Exacerbation of asthma is clearly an adverse health effect and we argue that individuals who constitute one‐tenth to one‐fifth of the general population, as well as a large part of the working population, should be taken into consideration.

## Conclusions

Our current comparison of published findings to information related to DNELs in the ECHA database of registered substances confirm our previous finding that there is a general lack of data on asthmatics. Nevertheless, we reveal that the available data on asthmatics are not used to the extent they could in the derivation of DNELs. Furthermore, several of the investigated acute/short‐term inhalation DNELs exceed or are close to the NOAEC and/or LOAEC values for asthmatics, indicating low or no safety margins. In conclusion, REACH registrants do not always consider the available data on asthmatics when setting acute/short‐term DNELs, this omission may result in values that are inappropriately high.

Data on human subjects, in particular high‐quality studies including susceptible subgroups can make a valuable contribution to health risk assessment and, if available, should be used for such purposes. In our opinion, the available data on asthmatics should be considered carefully, and if such data are lacking, this should be indicated explicitly in the case of respiratory irritants. In addition, it should be questioned why asthmatics are excluded as a group of interest in the setting of many, in particular occupational, exposure guideline values. Efforts towards developing guidance on how to identify and include susceptible subgroups in health risk assessments might help to improve the situation.

## Conflict of interest

GJ is a member of the SCOEL and has been a member of the NAS Subcommittee on AEGL. MÖ has been a member of the AEGL Advisory Committee. All other authors: none to declare.

## Supporting information

Supporting info item
