# Supplementary material for: Does industry take the susceptible subpopulation of asthmatic individuals into consideration when setting derived no‐effect levels?
Source: J Appl Toxicol. 2016 Jun 9;36(11):1379–91. doi: 10.1002/jat.3352 (PMC5089668; doi:10.1002/jat.3352)
Supplement: Supplementary file 1 — Supporting info item [file JAT-36-1379-s001.docx]

Supplemental material

Table S1. Overview of the 114 experimental studies on asthmatic subjects reviewed and our overall assessment of LOAECs and NOAECs for each substance.

| Substance | Exposure concentration (mg/m^3^) | Exposure duration (min) | Exposure conditions | Number of asthmatic subjects | Endpoint | LOAEC (mg/m^3^) | NOAEC  (mg/m^3^) | Comments | Reference |
| --- | --- | --- | --- | --- | --- | --- | --- | --- | --- |
| Acetaldehyde | Overall assessment |  |  |  |  | - | - | Several studies with subjects challenged with acetaldehyde for 2 min by tidal breathing to produce a 20% fall in FEV_1_ were available. These data were, however, considered insufficient for estimation of NOAEC and LOAEC. |  |
| Ammonia  7664-41-7 | 71 | 5-20 sec | Rest | 6 | Nasal airway resistance | 71 | - |  | *(McLean et al. 1979)* |
|  | 11.3-14.2 | 30 | Rest | 8 | FEV_1_,bronchial hyperreactivity | - | 11.3 - 14.2 | Effects defined either as a 20% decrease in FEV1 or one level reduction in the metacholine concentration required to induce bronchoconstriction. | *(Sigurdarson et al 2004)* |
| Ammonia | Overall assessment |  |  |  | Pulmonary function, bronchial hyperreactivity | - | 11.3 - 14.2 |  |  |
| Ammonium sulphate 7783-20-2 | 0.1, 1 | 16 | Rest | 17 | Flow at 40% and 60% of TLC on MEFV and PEFV curves | 1 | 0.1 |  | *(Utell et al 1982)* |
|  |  |  |  |  | SGaw, symptoms | - | 1 |  |  |
|  | 0.1 | 120 | Cycling for 15 of every 30 min. Workloads of 150-300 kg m/min. Doubled volume of ventilation per min. | 6 | FEV_1_, FVC, FEF_25-75%_, TLC, R_V_, R_T_ , symptoms | - | 0.1 |  | *(Avol et al. 1979)* |
|  | 0.07 | 40 | Light exercise on a treadmill during the last 10 min of exposure. | 9 (age 60-75) | FEV_1_, FVC, R_T_ | - | 0.07 |  | *(Koenig et al. 1993)* |
|  | 0.1 | 150 | Rest | 6 | Forced expiratory performance, lung volumes, respiratory resistance, symptoms | - | 0.1 | Aerosol size 3 um. | *(Hackney et al 1978)* |
|  | 0.1, 0.45, 1 | 16 | Rest | 17 | SGaw, FEV_1_, Vmax at 60% TLC, Vmax at 40% TLC on MEFV, 40 and 60% TLC on PEFV, Vtg, RV, TLC, FVC, symptoms | - | 1 |  | *(Utell et al. 1983)* |
|  | 0.5 | 60 | Rest | 14 | FEV_1_, SRaw | - | 0.5 | MMAD 7 µm LWC | *(Leduc et al 1995)* |
| Ammonium sulphate | Overall assessment |  |  |  | Pulmonary function | 1 | 0.5 |  |  |
| Chlorine  7782-50-5 | 1.16, 2.9 | 60 | Rest | 5 | FEV_1_, SRaw,  FEF_25-75%_ | 2.9 | 1.16 | Borderline statistically significant change in SRaw, FVC and PEFR among asthmatics compared to healthy subjects. Two out of four asthmatics showed symptoms at 2.9, but significance was not tested. | *(D'Alessandro et al. 1996)* |
| Diiron tris(sulphate) (Ferric sulfate)  10028-22-5 | 0.075 | 120 | Bicycle ergometer | 18 | FEV_1_, FVC, FEV_2_, MMFR, V_25-75%_, R_V_, TLV, R_T_, VC, symptoms. | - | 0.075 |  | *(Kleinman et al 1981)* |
| Formaldehyde  50-00-0 | 3.75 | 60 | 15 min on a bicycle ergometer twice. 37 L/min | 16 | FEV_1_, FVC, FEV_3_, FEF_25-75%_ and SGaw | - | 3.75 |  | *(Green et al. 1987)* |
|  |  |  |  |  | Symptoms of irritation | 3.75 | - |  |  |
|  | 2.5 | 30 | Moderate for 10 min. 450 kp m/min on a cycle ergometer. | 15 | PEFV, MEFV, TLC, VC, Raw, R_V_, FEV_1_, MEF_40%_, V_max50%_, symptoms of irritation | - | 2.5 |  | *(Witek et al. 1986)* |
|  | 1.24, 1.24, 3.72 | 10 | Rest | 7 | SRaw | - | 3.72 |  | *(Sheppard et al. 1984)* |
|  | 0.12, 0.85 | 90 | Rest | 15 | FEV_1_, Raw, functional residual capacity flow volume curves, symptoms of irritation | - | 0.85 |  | *(Harving et al. 1986, 1990)* |
|  | 3.72 | 180 | Rest | 9 | FEV_1_, FVC, _FEF25-27%_, SGaw, FRC, PD_35_SGaw | - | 3.72 |  | *(Sauder et al. 1987)* |
|  |  |  |  |  | Symptoms of irritation | 3.72 | - |  |  |
|  | 0.5 | 60 | Rest | 12 | FEV_1,_ FVC | - | 0.5 |  | *(Ezratty et al. 2007)* |
|  | 2.48 | 40 | Rest | 15 | FEV_1_, FVC, MEF_40%_ MEF_50%_, Raw, symptoms of irritation | - | 2.48 |  | *(Witek et al. 1987)* |
|  | 2.48 | 40 | Moderate (450 kpm/min) for 10 min | 15 | FEV_1_, FVC, MEF_40%_ MEF_50%_, Raw, symptoms of irritation | - | 2.48 |  |  |
| Formaldehyde | Overall assessment |  |  |  | Irritation | 3.7 | 2.48 |  |  |
| Hydrogen chloride 7647-01-0 | 1.19, 2.68 | 45 | 15 min walking on a treadmill twice. 2-3-fold increase in rest ventilation. | 10 | FEV_1_, FVC, R_T_, V_max50_ , V_max75_, peak flow, nasal work, symptoms of irritation | - | 2.68 |  | *(Stevens et al 1992)* |
| Hydrogen sulphide  7783-06-4 | 2.8 | 30 | Rest | 10 | FEV_1_, FVC, FEF_25-75%_, Raw, SGaw, FVC | - | 2.8 |  | *(Jäppinen et al 1990)* |
| Nitric acid  7697-37-2 | 0.13 | 40 | Last 10 min; Moderate on a treadmill, mean ventilation 31.8±10.2 L/min (~4 times higher than during rest). | 5 | FEV_1_, RT  Symptoms | 0.13  - | -  0.13 |  | *(Koenig et al 1989a)* |
| Nitric acid | Overall assessment |  |  |  | Pulmonary function | 0.13 | - |  |  |
| Sodium hydrogen sulphate (Sodium bisulfate)  7681-38-1 | 0.1, 1 | 16 | Rest | 17 | FEV_1._ SGaw, symptoms of irritation | - | 1 |  | *(Utell et al 1982)* |
|  |  |  |  |  | Flow at 40% and 60% of TLC on MEFV and PEFV curves | 1 | 0.1 |  |  |
|  | 0.1, 0.450, 1 | 16 | Rest | 17 | FEV_1_, SGaw or V_max_ 40-60% at TLC | - | 1 |  | *(Utell et al 1984)* |
|  | 0.1, 0.45, 1 | 16 | Rest | 17 | SGaw, FEV_1_ Vmax at 60% TLC, Vmax at 40% TLC on MEFV, 40 amd 60% TLC on PEFV, Vtg, R_V_, TLC, FVC, symptoms | - | 1 |  | *(Utell et al. 1983)* |
| Sodium hydrogen sulphate (Sodium bisulfate) | Overall assessment |  |  |  | Pulmonary function | 1 | 0.45 |  |  |
| Sodium nitrate  7631-99-4 | 7 | 16 | Rest | 11 | SGaw, V_max_, 40-60% TLC on MEFV or PEFV, R_V_, TGV, TLC, FEV_1_, FVC or symptoms | - | 7 |  | *(Utell et al 1979)* |
|  | 1, 2, 3 | 10 | Rest | 5 | FEV_1_, R_RS_, vital capacity | - | 3 |  | *(Sackner et al 1981)* |
|  | 3 | 10 | Rest | 6 | Spirometry, plethysmography, respiratory mechanics, breath diffusing capacity | - | 3 |  |  |
| Sodium nitrate | Overall assessment |  |  |  | Pulmonary function | - | 7 |  |  |
| Sulphur dioxide  7446-09-5 | 0.715, 1.43, 2.145, 2.86 | 40 | Bicycle. 450 kp m/min during the first 10 min. Ventilation 35 L/min. | 10 | FEV_1_, MEF_40%_ | 2.145 | 1.43 |  | *(Schacter et al. 1984) (Witek et al 1985b)* |
|  |  |  |  |  | Symptoms | 2.145 | 1.43 |  |  |
|  |  |  |  |  | Raw | 2.86 | 2.145 |  |  |
|  |  |  |  |  | V_max50%_ | 1.43 | 0.715 |  |  |
|  | 1.43 | 10 (tidal breathing) + 10 (hyperventilation) | 10 min isocapnic hyperventilation at 30 L/min. | 46 | SRaw | 1.43 | - |  | *(Magnusson et al. 1990)* |
|  | 2.86, 8.58, 14.3 | 10 | rest | 7 | SRaw | 2.86 | - |  | *(Sheppard et al 1980)* |
|  |  |  |  |  | Symptoms | - | 14.3 |  |  |
|  | 0.715, 1.43, 2.145, 2.86 | 30 | Moderate exercise for 10 min. 450 kp m/min on a cycle ergometer. |  | FEV_1_, Raw, MEF_40%_ | 2.145 | 1.43 |  | *(Witek et al. 1986)* |
|  |  |  |  |  | PEFV, MEFV, TLC, VC, R_V_ or V_max50%_., symptoms | - | 2.86 |  |  |
|  | 0.57, 1.14, 1.72 | 60 | Alternating 10 min exercise. Ergometer. Oronasal breathing. 40 L/min. | 16 | SRaw, FEV_1_, symptoms | - | 1.72 |  | *(Linn et al 1987)* |
|  | 22.88 | 5 | Rest | 4 | Raw, SGaw | 22.88 | - |  | *(Snashall et al 1982)* |
|  |  |  |  |  | Vtg | - | 22.88 |  |  |
|  | 0.57 | 60 | Rest | 12 | FEV_1_, FVC, MMEF, symptoms. | - | 0.57 |  | *(Tunnicliffe et al 2001) (Tunnicliffe et al 2003)* |
|  | 1.43 | 180 | Rest | 40 | MMFR | 1.43 | - |  | *(Jaeger et al 1979)* |
|  |  |  |  |  | FEV_1_, VC, FRC, FVC, Raw, R_V_, symptoms | - | 1.43 |  |  |
|  | 0.57, 1.14, 1.72 | 5 | Heavy workload. Bicycle 5 min. 730 kgm/min and 48L/min | 23 | Symptoms | 0.57 | - |  | *(Linn et al. 1983 b)* |
|  |  |  |  |  | SRaw, V_max25_, V_max50_, V_max75_ | 1.14 | 0.57 |  |  |
|  |  |  |  |  | FEV_1_, FEV_2_, FEV_3_, FVC, PEFR | 1.72 | 1.14 |  |  |
|  | 0.715, 1.43, 2.86 | 75 | Treadmill. 10 min intervals. 42.4L/min. | 28 | SRaw, TGV | 1.43 | 0.715 |  | *(Roger et al. 1985)* |
|  |  |  |  |  | FEV_1_/FVC, FEF_25-75_, FEF_50_, symptoms | 2.86 | 1.43 |  |  |
|  | 1.43 | 30 (tidal breathing) | Rest | 14 | SRaw and PV_100_SRaw | - | 1.43 |  | *(Jörres et al. 1990)* |
|  | 0.715 | 10 | Bicycle. 5 min intervals. 750 kgm/min. | 19 | SRaw | 0.715 | - |  | *(Bethel et al. 1985)* |
|  | 2.15 | 180 | 10min in the beginning. 40 L/min. | 17 | SRaw, FEV_1_, symptoms | 2.15 | - | Symptoms not statistically significant. | *(Hackney et al. 1984)* |
|  | 1.43 | 50 | 20 min at the end on treadmill. 43.3 L/min. | 10 | FEV_1_, R_T_, FRC, V_max50%,_ V_max75%_ | 1.43 | - | Exposure via mouthpiece. | *(Koenig et al. 1985)* |
|  |  |  |  |  | FEV1, RT, Vmax50%, Vmax75% | 1.43 | - | Exposure via face mask. |  |
|  |  |  |  |  | FRC | - | 1.43 | Exposure via face mask. |  |
|  | 1.43 | 5 | Moderately heavy work rate. 5 min on bicycle. 750 kgm/min. | 10 | SRaw | 1.43 | - |  | *(Bethel et al. 1983a)* |
|  | 1.43 | 5 | Moderately heavy work rate. 5 min on bicycle. 250, 500 or 750 kgm/min. | 9 | SRaw | 1.43 | - | Mouthpiece: Significant bronchoconstriction at moderate and high workloads. Facemask: only high workloads resulted in significant bronchoconstriction. | *(Bethel et al. 1983 b)* |
|  | 2.15 | 10 | 10 min on bicycle. 650 kgm/min. 40 L/min. | 23 | SRaw, TGV | 2.15 | - | The excess increase in SRaw was significantly greater with mouthpiece compared to free breathing. | *(Linn et al. 1983a)* |
|  |  |  |  |  | FEV_1_, symptoms | - | 2.15 |  |  |
|  | 1.72 | 360 | Bicycle 5 min in beginning and 5 min after 300 min. 50 L/min. | 14 | SRaw, SGaw, | 1.72 | - |  | *(Linn et al. 1984a)* |
|  |  |  |  |  | Vtg, symptoms | - | 1.72 |  |  |
|  | 1.72 | 5 | 5 min of heavy exercise on a bicycle ergometer at a ventilation rate of 50 L/min. | 22 | SRaw, SGaw, Vtg, symptoms | 1.72 | - |  | *(Linn et al. 1985)* |
|  | 1.43, 2.86 | 10 | Light, medium and heavy exercise on a treadmill. Mean ventilation rates of 30, 36 and 43 L/min. Eight different physical tasks each lasting 10 min.  . | 14 | FEV_1_, SRaw, symptoms | 1.43 | - | Light, medium and heavy exercise. | *(Gong et al. 1995)* |
|  | 1.43 | 3 | Rest | 8 | SRaw | 1.43 | - | Eucapnic hyperpnea. | *(Sheppard et al. 1983)* |
|  | 1.43, 2.86 | 40 | 10 min on a treadmill. 5-6-fold increase in mean resting ventilation. | 9 | R_T_, FEV_1_, V_max50%,_ V_max75%_ | 1.43 | - | Similar effects were seen after nasal inhalation, but the changes did not differ significantly from baseline. | *(Koenig et al. 1983)* |
|  |  |  |  |  | FRC, symptoms | - | 1.43 |  |  |
|  | 2.86 | 60 + 30 | Three sessions of 10-min on a treadmill. 41 L/min + 30 min treadmill. 22 L/min. | 10 | SRaw, FEV_1_/ FVC, FEF_25-75%_, FEV_1_ | 2.86 | - |  | *(Kehrl et al. 1987)* |
|  | 1.43 | 60 | Rest | 9 | V_max 50%_, V_max75%_ | 1.43 | - |  | *(Koenig et al. 1980)* |
|  |  |  |  |  | FEV_1_, R_T_, FRC, symptoms | - | 1.43 |  |  |
|  | 2.86 | 30 sec, 1, 2, 5 | Treadmill 5 min before and during exposure. 40 L/min. | 12 | SRaw, symptoms | 2.86 |  | After 2 and 5 min exposure. Not after 0.5 and 1 min. | *(Horstman et al. 1988)* |
|  | 1.43 | 3 | Rest | 7 | SRaw, symptoms | - | 1.43 | Statistically significant decrease at -10.6 ºC but not at 23.3 ºC. | *(Bethel et al 1984)* |
|  | 0.57 | 360 | Rest | 10 | FEV_1_, FVC, PD_20_ FEV_1_ | - | 0.57 |  | *(Devalia et al. 1994)* |
|  | 0.286 | 60 | Two 15-min intervals on treadmill. 30L/min. | 13 (age 12-18) | FEV_1_, R_T_, V_max50%,_, symptoms | - | 0.286 |  | *(Koenig et al 1990)* |
|  | 2.86, 5.72, 11.44, 22.88 | 4 (eucapnic hyperventilation) | Eucapnic hypernea 20 L/min. | 12 | PC_8_SRaw, FEV_1_≥70% | 4.29 | - | Effects of zafirlukast (medication) on SO_2_-related responses. Placebo+SO_2_ | *(Lazarus et al 1987)* |
|  | 0.57, 1.14, 1.72 | 5 | 5 min on bicycle. 48 L/min | 23 | FEV_1_, FEV_2_, FEV_3_, PEFR, Vtg | 1.72 | 1.14 |  | *(Linn et al 1977)* |
|  |  |  |  |  | SRaw, V_max25%,_ V_max50%,_,V_max75%_ | 1.14 | 0.57 |  |  |
|  |  |  |  |  | Symptoms | 0.57 | - |  |  |
|  | 0.715, 1.43 | 60 | Three 10-min sessions on bicycle. 27 L/min. | 24 | SRaw, Raw, FVC, FEV_1_, Vtg, symptoms | - | 1.43 |  | *(Linn et al 1982)* |
|  | 1.72 | 5 | 5 min. 50 L/min. | 24 | SRaw, SGaw, symptoms | 1.72 |  |  | *(Linn et al 1984b)* |
|  | 0.86, 1.72 | 5 | 5 min bicycle. 50 L/min. | 24 | SRaw, SGaw, Vtg, symptoms | - | - | Statistics not presented for exposure to SO_2_ and air at 21 ºC. Only a picture. | *(Linn et al 1984c)* |
|  | 0.86, 1.72 | 10 | 10 min bicycle. 50 L/min. | 20 | FEV_1_, FVC, SRaw, Symptoms | - | - | Effects of metaproterenol sulfate on SO_2_-related responses. Statistics for air+SO_2_ compared to baseline not presented. | *(Linn et al 1988)* |
|  | 0.86, 1.72 | 10 | 10 min bicycle. 50 L/min. | 21 | SRaw, FEV_1_, symptoms | - | - | Effects of metaproterenol on SO_2_-related responses. SO_2_ exposure of untreated subjects not performed. | *(Linn et al 1990)* |
|  | 0.286, 0.715, 1.43, 2.86 | 10 + 10 | Bicycle 10 min. 400 kpm/min + Bicycle 10 min. 350 kpm/min, 30 L/min. | 7 + 6 | SRaw | 0.715 | 0.286 |  | *(Sheppard et al 1981a)* |
|  | 1.43, 2.86 | 10 | Bicycle 10 min. 38 L/min. | 6 | SRaw | 1.43 | - | Effects of cromolyn on SO_2_-related responses. Statistics for placebo+SO_2_. | *(Sheppard et al 1981b)* |
|  | 0.715, 1.43, 2.15, 2.86 | 40 | Bicycle 10 min. 450 kpm/min. | 8 | MEF_40%_ | 2.15 | 1.43 |  | *(Witek et al 1985a)* |
|  | 2.15 | 10 | Bicycle 10 min. 35 L/min. | 12 | FEV_1_, SRaw, Symptoms, eosinophils | 2.15 | - | Effects of montelukast (medication) on SO_2_-related responses. Placebo+SO_2_. | *(Gong et al 2001)* |
|  | 2.15 | 10 | Bicycle 10 min. 29 L/min. | 10 | FEV_1_, Symptoms | 2.15 | - | Effects of salmeterol on SO_2_-related responses. Placebo+SO_2_. 10% or greater loss in FEV_1_. Numerous symptoms. | *(Gong et al 1996)* |
|  | 1.43 | 5 | Bicycle 5 min. 550 kpm/min. | 6 | SRaw | 1.43 | - | Symptoms. No statistical analyses. | *(Kirkpatrick et al 1982)* |
|  | 2.15 | 10 | Treadmill 10 min. 4- to 5-fold increase in minute ventilation. | 10 | R_T_, FEV_1_ | 2.15 | - | Effects of albuterol on SO_2_-related responses. Placebo+SO_2_. | *(Koenig et al 1987)* |
|  |  |  |  |  | FRC, symptoms | - | 2.15 |  |  |
|  | 2.86 | 10 | Treadmill 10 min. 35±12.1 L/min | 8 | FEV_1_, R_T_ | 2.86 | - | Allergic subjects with exercise-induced bronchospasms. Effects of cromolyn on SO_2_-related responses. Placebo+SO_2_. | *(Koenig et al 1988)* |
|  | 2.86 | 10 | Treadmill 10 min. 21.6 L/min. | 8 | FEV_1_, R_T_ | 2.86 | - | Effects of theophylline on SO_2_-related responses. Statistics not presented for placebo+SO_2_ compared to placebo+air or baseline. 16% decrease in FEV_1_ and 37% increase in RT. | *(Koenig et al 1992)* |
|  | 1.43, 2.86 | 30 | Treadmill during the last 10 min. 26.4 L/min | 9 | FEV_1_, SR_T_, V_max50%,_ V_max75%_ | 2.86 | 1.43 | SR_T_ = specific total respiratory resistance | *(McManus et al 1989)* |
|  |  |  |  |  | Symptoms | - | 2.86 |  |  |
|  | 1.43 | 10 | Treadmill 10 min. 3-fold increase in resting ventilation. | 47 | FEV_1_, FVC, FEF_25-75%_ and PEF | 1.43 | - | Statistics not presented for separate responders and non-responders. Non-responders had decrease -10%, -5.6%, -17.3%, -9.1%. More symptoms among responders. | *(Trenga et al 1999)* |
|  | 2.15 | NA (isocapnic hyperventilation) | Hyperventilation 23 L/min | 25 | PV_75_SRaw (75% increase) | 2.15 | - | Effects of salbutamol on SO2-related responses. No exposure to SO2 alone except for in a preliminary test. | *(Wiebicke et al 1990)* |
|  | 1.43 | 10 | Treadmill 10 min. 3-fold increase in rest ventilation. | 62 | FEV_1_, FVC, PEFR and FEF_25-75%_ | - | - | Statistics were not presented. All responders had wild type allelle TNF-α (TNF-1). Suggests that this wildtype has an association with asthma. 13 of 62 had FEV1 decrease of 13% | *(Winterton et al 2001)* |
|  | NA | 3 (eucapnic hyperventilation) | Rest | 10 | NA | NA | NA | “Potential bronchoconstrictor stimuli in acid fog” | *(Balmes et al 1989b)* |
|  | 2.86 | 20 | Treadmill the last 10 min. Walked 2 mph. | 22 | FEV_1_, R_T_, V_max50%_ | 2.86 | - | Statistically significant changes in Vmax50, RT and FEV1 after SO2 compared to baseline. | *(Heath et al 1994)* |
|  |  |  |  |  | White blood cells, epithelial cells, symptoms | - | 2.86 | No statistically significant changes in precentages of any cells or reported symptoms. |  |
|  | 2.86 | 40 | Treadmill the last 10 min. Six-fold increase in resting ventilation. | 8 (age 14-18) | FEV_1_, R_T_, V_max50%,_ V_max75%_ | 2.86 | - |  | *(Koenig et al 1981)* |
|  |  |  |  |  | FRC, Symptoms | - | 2.86 |  |  |
|  | 0.286 | 40 | Last 10 min; Moderate on a threadmill mean ventilation 31.8±10.2 L/min (~4 times higher than during rest). | 5 (age 12-18) | FEV_1_, R_T_, symptoms | - | 0.286 |  | *(Koenig et al 1989)* |
|  | 1.43, 2.86, 5.72, 11.44 | 4 (eucapnic hyperventilation) | Rest | 4 | Nasal resistance, nasal symptoms | - | 11.44 | Nasal mask. | *(Tam et al 1988)* |
|  | 1.43, 2.86, 5.72 | 4 (eucapnic hyperventilation) | Eucapnic hypernea 20 L/min. | 8 | PC_8unit_SRaw, Symptoms, Nasal resistance, nasal symptoms | - | 5.72 | Nasal mask. |  |
|  | 1.43, 2.86, 5.72 | 4 (eucapnic hyperventilation) | Eucapnic hypernea 20 L/min. | 8 | PC_8unit_SRaw, Symptoms | 5.72 | 2.86 | Mouthpiece. |  |
|  | 1.06 | 120 | 15 min of each 30 min to increase min ventilation 2-2.5 fold. | 4 | FEV_1_, FVC | - | 1.06 |  | *(Bell et al 1977)* |
|  | 0.715, 1.43, 2.86 | 75 | Treadmill 10 min intervals. 21 l/min/m2 BSA | 20 | SRaw | 1.43 | 0.715 |  | *(Kehrl et al 1983)* |
|  |  |  |  |  | FVC, FEV_1_, Raw, IGV | - | 0.715 |  |  |
| Sulphur dioxide | Overall assessment |  |  |  | Pulmonary function, FEV_1_ | Average  2.21  Median 2.15 | Average  1.16  Median 1.43 |  |  |
| Sulphuric acid  7664-93-9 | 0.38, 1.06, 1.5 | 60 | Three 10-min periods. 50 L/min | 21 | FEV_1_, FVC | 1.06 | 0.38 |  | *(Avol et al. 1988b)* |
|  |  |  |  |  | SRaw | - | 1.5 |  |  |
|  |  |  |  |  | Symptoms | 1.06 | 0.38 |  |  |
|  | 0.150, 0.680 | 60 | 10 min intervals. | 6 | SRaw | 0.680 | 0.15 |  | *(Linn et al 1986b)* |
|  | 0.1, 1 | 16 | Rest | 17 | FEV_1_, SGaw, 60% TLC on MEFV, 40-60% TLC on PEFV | 1 | 0.1 |  | *(Utell et al 1982)* |
|  |  |  |  |  | Symptoms | - | 1 |  |  |
|  | 0.1, 0.450, 1 | 16 | Rest | 17 | FEV_1_ | 1 | 0.45 |  | *(Utell et al 1984)* |
|  |  |  |  |  | V_max 40-60%_ at TLC | - | 1 |  |  |
|  |  |  |  |  | SGaw | 0.45 | 0.1 |  |  |
|  | 0.1 | 356 | Six 50-min periods. 29 L/min. | 30 | FEV_1_, SRaw, Symptoms | - | 0.1 |  | *(Linn et al. 1994)* |
|  | 0.1 | 120 | Cycled 15 of every 30 min. 150-300 kg m/min. Doubled minute volume of ventilation | 6 | FEV_1_, FVC, FEF_25-75%_, TLC, R_V_, R_T_, symptoms | - | 0.1 |  | *(Avol et al. 1979)* |
|  | 0.1 | 180 | 10 min of every 30 min to at least quadruple minute ventilation. | 30 | FEV_1_, SGaw, FVC, symptoms | - | 0.1 |  | *(Frampton et al. 1995)* |
|  | 0.1 | 60 | 10-min periods on bicycle. 50 L/min. | 15 | FEV_1_, SRaw, FVC, symptoms. | - | 0.1 |  | *(Anderson et al. 1992)* |
|  | 0.5, 1, 2 | 60 | 10-min periods on bicycle. 50 L/min. | 22 | PEFR , Symptoms | 2 | 1 |  | *(Avol et al 1988a)* |
|  |  |  |  |  | FEV_1_, FVC, Vtg or SRaw | - | 2 |  |  |
|  | 0.07 | 40 | Light exercise on a treadmill during the last 10 min of exposure. | 9 (age 60-75) | FEV_1_, FVC or R_T_ | - | 0.07 |  | *(Koenig et al. 1993)* |
|  | 0.01, 0.1, 1 | 10, 10, 10 | Rest | 5, 6, 12 | FEV_1_, VC, R_T_, ERV, R_V_, TLC, PEFR, V_max50-75%,_ Raw, SGaw, symptoms. | - | 1 |  | *(Sackner et al. 1978)* |
|  | 0.075, 0.1, 0.35, 0.45, 1 | 16, 30, 120, 240 | 16 min, all conc: rest,  30 min, 0.35 mg/m^3^: 10 min exercise  120 min, 0.075 mg/m^3^: 10 min exercise,four times  240 min, 0.1, 0.45 mg/m^3^: 10 min exercise, three times | 17 | FEV_1_, SGaw | 1 | 0.45 |  | *(Utell et al. 1991)* |
|  | 0.1 | 180 | 10 min of every 30 min. Quadrupled the expired volume of ventilation/min (60-200 watts). | 30 | FEV_1_, FVC, SGaw, symptoms | - | 0.1 |  | *(Utell et al. 1994)* |
|  | 0.2, 2 | 60 | Rest | 12 | FEV_1_, FVC, MMEF or symptoms | - | 2 |  | *(Tunnicliffe et al 2003)* |
|  | 0.075 | 150 | 300 kgm/min for fit people, 150 for others. Alternating 15 min with rest. 4 times. | 6 | Forced expiratory performance, lung volumes, respiratory resistance, symptoms | - | 0.075 | Aerosol size 3 um. 2/6 showed possibly significant changes in respiratory resistance | *(Hackney et al 1978)* |
|  | 0.075 | 120 | Cycling 10 min after 10, 35, 60 and 90 min of exposure. Quadrupled minute ventilation. | 8 (age 54-70) | FVC, FEV_1_, SGaw | - | 0.075 | Fell after exercise, but small differences between air and acid. | *(Bauer et al. 1988)* |
|  | 2.8 ± 0.8 | 16 (tidal breathing) | Rest | 11 | SRaw, symptoms | - | 2.9 | Large particle, 6.1 µm | *(Aris et al. 1991)* |
|  | 2.9 ± 0.9 | 16 (tidal breathing) | Rest | 11 | SRaw, symptoms | - | 2.9 | Small particle, 0.4 µm |  |
|  | 3.02 ± 0.5 | 16 (tidal breathing) | Rest | 9 | SRaw, symptoms | - | 3.02 | Large particle, 5.8 µm |  |
|  | 3.37 ± 0.62 | 16 (tidal breathing) | Rest | 9 | SRaw, symptoms | - | 3.37 | Small particle, 0.4 µm |  |
|  | 2.97 ± 0.86 | 16 (tidal breathing) | Rest | 6 | SRaw, symptoms | - | 2.97 | Small particle, 0.4 µm |  |
|  | 0.96 ± 0.2 | 16 (tidal breathing) | Cycling 15-min intervals. 100 W. | 10 | SRaw, symptoms | - | 0.96 | Large particle, 6.4 µm  Low liquid-water-content (LWC) |  |
|  | 1.40 ± 0.3 | 16 (tidal breathing) | Cycling 15-min intervals. 100 W. | 10 | SRaw, symptoms | - | 1.4 | Large particle, 6.6 µm High LWC |  |
|  | 0.1, 1 | 60 | Rest | 13 | FEV_1_ | 1 | 0.1 |  | *(Tunnicliffe et al. 2001)* |
|  | 0.046 ± 0.011  0.127 ± 0.021 | 60 | Cycling 10 min. 20 L/min/m^2^. | 30 (age 8-16) | FEV_1_, SRaw, Symptoms | - | 0.127 |  | *(Avol et al. 1990)* |
|  | 0.051-0.176 | 45 | The last 10 min, moderate exercise. | 14 (age 12-19) | FEV_1_, FVC | 0.051-0.176 | - |  | *(Hanley et al. 1992)* |
|  |  |  |  |  | V_max50_, V_max75_ , R_T_, symptoms | - | 0.051-0.176 |  |  |
|  | 0.1 | 40 | Treadmill 10min. 5-6-fold increase in mean resting minute ventilation. | 10 (age 14-18) | RT, Vmax50, Vmax75 and FEV_1_. | 0.1 | - | Exposure via mouthpiece | *(Koenig et al. 1985)* |
|  |  |  |  |  | FRC | - | 0.1 |  |  |
|  | 0.1 | 40 | Treadmill 10 min. 5-6-fold increase in mean resting minute ventilation. | 10 (age 14-18) | RT, V_max50_, V_max75_ and FEV_1_. | 0.1 | - | Exposure via face mask |  |
|  |  |  |  |  | FRC | - | 0.1 |  |  |
|  | 0.1 | 40 | Treadmill 10 min. 5-6-fold increase in mean resting minute ventilation. | 10 (age 12-17) | RT, V_max50_, V_max75_ and FEV_1_. | 0.1 | - |  | *(Koenig et al. 1983)* |
|  |  |  |  |  | Symptoms | - | 0.1 |  |  |
|  | 0.1 | 40 | Rest | 10 (age 12-17) | RT, V_max50_, V_max75_, FEV_1_ and symptoms | - | 0.1 |  |  |
|  | 0.068 | 40 | Last 10 min; Moderate on a treadmill, mean ventilation 31.8±10.2 L/min (~4 times higher than during rest). | 5 (age 12-18) | FEV_1_ | 0.068 | - |  | *(Koenig et al. 1989)* |
|  |  |  |  |  | R_T_, symptoms | - | 0.068 |  |  |
|  | 0.035, 0.07 | 45 | Treadmill 15-min intervals. Tripled resting minute ventilation. | 14 | FEV_1_ | 0.035 | - |  | *(Koenig et al. 1992a) (Koenig et al 1992b)* |
|  |  |  |  |  | FVC, RT | - | 0.07 |  |  |
|  | 0.035, 0.07 | 90 | Treadmill 15-min intervals. Tripled resting minute ventilation. | 14 | FEV_1_, FVC, RT | - | 0.07 |  |  |
|  | 0.5 | 60 | Rest | 14 | FEV_1_, SRaw, FRC, R_V_, ERV, TLC | - | 0.5 |  | *(Leduc et al. 1995)* |
|  | 0.122, 0.242, 0.410 | 60 | Cycling 10-min intervals. 42 L/min. | 27 | FEV_1_, SRaw, FVC, Vtg, MMFR, Symptoms | - | 0.410 |  | *(Linn et al. 1986)* |
|  | 0.1, 0.3, 1 | 60 | Rest | 10 | FEV_1_, SGaw, MMEF, V_25_ | 1 | 0.3 |  | *(Spektor et al. 1985)* |
|  | 0.1, 0.45, 1 | 16 | Rest | 16 | FEV_1_, Vmax at 60% TLC, Vmax at 40% TLC on MEFV, 40 amd 60% TLC on PEFV | 1 | 0.45 |  | *(Utell et al. 1983)* |
|  |  |  |  |  | SGaw | 0.45 | 0.1 |  |  |
|  |  |  |  |  | Vtg, R_V_, TLC, FVC, Symptoms | - | 1 |  |  |
|  | 0.35 | 30 | Cycling last 10 min 300 kp m/min. 3 fold or greater increase in minute ventilation. | 15 | FEV_1_, max expiratory flow rates at 60% total lung capacity | 0.35 | - | Also in FVC and SGaw compared to baseline but not compared to NaCl. | *(Utell et al. 1989)* |
|  | 0.1, 0.25, 0.5 | 60 | 10-min intervals. 42 L/min. | 27 | FEV_1_, SRaw, Symptoms | - | 0.5 |  | *(Linn et al 1985)* |
|  | 0.046±0.011  0.127±0.021 | 40 | Last 10 min. 20L/min | 32 (age 8-16) | SRaw, Forced expiratory function, Symptoms | - | 0.127 |  | *(Linn et al 1990)* |
|  | 0.1, 0.45, 0.1, 0.45 | 60, 60 | 10 min. 300 kpm/min. Tripled minute ventilation. | 11 | SGaw | 0.45 | 0.1 | Chamber. | *(Utell et al 1988)* |
|  |  |  |  |  | FEV_1_, FVC | - | 0.45 |  |  |
|  | 0.1, 0.45, 0.1, 0.45 | 16, 16 | Rest | 17 | SGaw | 0.45 | 0.1 | Mouthpiece wearing noseclip. |  |
|  |  |  |  |  | FEV_1_, FVC | - | 0.45 |  |  |
|  | 1 | 60 | Cycling 15-min intervals. 100 W. | 10 | SRaw, Symptoms | - | 1 | One exposure in chamber and one in mouthpiece wearing noseclip. | *(Aris et al. 1990)* |
| Sulphuric acid | Overall assessment |  |  |  | Pulmonary function, FEV_1_ | Average: 0.55 Median: 0.45 | Average: 0.42 Median: 0.21 |  |  |
| 4-methyl-m-phenylene 584-84-9 (80% 2,4-Toluene diisocyanate and 20% 2,6-Toluene diisocyanate) | 0.071, 0.142 | 120 | Rest | 15 | FEV_1_, IGV, SRaw, Raw, VC, symptoms | - | 0.142 |  | *(Baur 1985)* |

The experimental studies documented in Appendix Table S1 (n = 114).

**ACETALDEHYDE**

Several studies with subjects challenged with acetaldehyde for 2 min by tidal breathing to produce a 20% fall in FEV_1_ were available. These data were considered insufficient for estimation of NOAEC and LOAEC

**AMMONIA**

McLean JA, Mathews KP, Solomon WR, Brayton PR, Baynel NK. 1979. Effect of ammonia on nasal resistance in atopic and nonatopic subjects. Ann. Otol. Rhinol. Laryngol. 88(2 Pt.1):228-234.

Sigurdarson ST, O’Shaughnessy PT, Watt JA, Kline JN. 2004. Experimental human exposure to inhaled grain dust and ammonia: Towards a model of concentrated animal feeding operations. Am. J. Ind. Med. 46(4):345-348

**AMMONIUM SULPHATE**

Avol EL, Jones MP, Bailey RM, Chang NM, Kleinman MT, Linn WS, Bell KA, Hackney JD. 1979. Controlled exposures of human volunteers to sulfate aerosols. Health effects and aerosol characterization. Am. Rev. Respir. Dis. 120(2):319-327.

Hackney JD, Linn WS, Bell KA. 1978. Experimental Studies of the Human Health Effects of Sulfur Oxides. Bull. N. Y. Acad. Med. 54(11):1177–1185.

Koenig JQ, Dumler K, Rebolledo V, Williams PV, Pierson WE. 1993. Respiratory effects of inhaled sulfuric acid on senior asthmatics and nonasthmatics. Arch. Environ. Health. 48(3):171-175

Leduc D, Fally S, de Vuyst P, Wollast R, Yernault J-C. 1995. Acute exposure to realistic acid fog: effects on respiratory function and airway responsiveness in asthmatics. Environ. Res. 71:89-98

Utell MJ, Morrow PE, Hyde RW. 1982. Comparison of Normal and Asthmatic Subjects Responses to Sulfate Pollutant Aerosols. Ann. Occup. Hyg. 26:691-697

Utell MJ, Morrow PE, Speers DM, Darling J, Hyde RW. 1983. Airway responses to sulfate and sulfuric acid aerosols in asthmatics. An exposure-response relationship. Am. Rev. Respir. Dis. 128:444-450

**CHLORINE**

D´Alessandro A, Kuschner W, Wong H, Boushey HA, Blanc PD. 1996. Exaggerated responses to chlorine inhalation among persons with nonspecific airway hyper reactivity. Chest. 109:331-337.

**DIIRON TRIS(SULPHATE)**

Kleinman MT, Linn WS, Bailey RM, Anderson KR, Whynot JD, Medway DA, Hackney JD. 1981. Human exposure to ferric sulfate aerosol: effects on pulmonary function and respiratory symptoms. Am. Ind. Hyg. Assoc J. 42(4):298-304

**FORMALDEHYDE**

Ezratty V, Bonay M, Neukirk C, Orset-Guillossou G, Dehoux M, Koscielny S, Cabanes PA, Lambrozo J, Aubier M. 2007. Effect of formaldehyde on asthmatic response to inhaled allergen challenge. Environ. Health Persp. 115:210-214

Green DJ, Sauder LR, Kulle TJ, Bascom R. 1987. Acute response to 3.0 ppm formaldehyde in exercising healthy nonsmokers and asthmatics. Am. Rev. Respir. Dis. 135:1261-1266.

Harving H, Korsgaard J, Dahl R, Pedersen OF, Molhave L. 1986. Low concentrations of formaldehyde in bronchial asthma: a study of exposure under controlled conditions. Brit. Med. J. 293:310.

**Same data presented in Harving et al., 1986**: Harving H, Korsgaard J, Pedersen OF, Molhave L, Dahl R. 1990. Pulmonary function and bronchial reactivity in asthmatics during low-level formaldehyde exposure. Lung. 168:15-21.

Sauder LR, Green DJ, Chatham MD, Kulle TJ. 1987. Acute pulmonary response of asthmatics to 3.0 ppm formaldehyde. Toxicol. Ind. Health. 3:569-577

Sheppard D, Eschenbacher WL, Epstein J. 1984. Lack of bronchomotor response to up to 3 ppm formaldehyde in subjects with asthma. Environ. Res. 35:133-139

Witek TJ, Schacter EN, Tosun T, Leaderer BP, Beck GJ. 1986. Controlled human studies on the pulmonary effects of indoor air pollution: experiences with sulfur dioxide and formaldehyde. Environ. Intl. 12:129-135.

Witek TJ, Schacter EN, Tosun T, Beck GJ, Leaderer BP. 1987. An evaluation of respiratory effects following exposure to 2.0 ppm formaldehyde in asthmatics: lung function, symptoms, and airway reactivity. Arch. Environ. Health 42:230-237

**HYDROGEN CHLORIDE**

Stevens B, Koenig JQ, Rebolledo V, Hanley QS, Covert DS. 1992. Respiratory effects from the inhalation of hydrogen chloride in young adult asthmatics. J. Occup. Med. 34:923-929.

**HYDROGEN SULPHIDE**

Jäppinen P, Vilkka V, Marttila O, Haahtela T. 1990. Exposure to hydrogen sulfide and respiratory function. Br. J. Ind. Med. 47:824-828

**NITRIC ACID**

Koenig JQ, Covert DS, Pierson WE. 1989a. Effects of inhalation of acidic compounds on pulmonary function in allergic adolescent subjects. Environ. Health. Persp. 79:173-178.

**SODIUM HYDROGEN SULPHATE**

Utell MJ, Morrow PE, Hyde RW. 1982. Comparison of Normal and Asthmatic Subjects Responses to Sulfate Pollutant Aerosols. Ann. Occup. Hyg. 26:691-697

Utell MJ, Morrow PE, Speers DM, Darling J, Hyde RW. 1983. Airway responses to sulfate and sulfuric acid aerosols in asthmatics. An exposure-response relationship. Am. Rev. Respir. Dis. 128:444-450

Utell MJ, Morrow PE, Hyde RW. 1984. Airway reactivity to sulfate and sulfuric acid aerosols in normal and asthmatic subjects. J. Air. Pollut. Control. Assoc. 34:931-935

**SODIUM NITRATE**

Sackner MA, Ford D. 1981. Effects of breathing nitrate aerosols in high concentrations for 10 minutes on pulmonary function in normal and asthmatic adults, and preliminary results in normal exposed to nitric acid fumes. Am. Rev. Resp. Dis. 123:151

Utell MJ, Swinburne AJ, Hyde RW, Speers DM, Gibb FR, Morrow PE. 1979. Airway reactivity to nitrates in normal and mild asthmatic subjects. J. Applied. Physiol. 46(1):189-196

**SULPHUR DIOXIDE**

Balmes JR, Fine JM, Gordon T, Sheppard D. 1989b. Potential bronchoconstrictor stimuli in acid fog. Environ. Health. Perspect. 79:163-166

Bell KA, Linn WS, Hazucha M, Hackney JD, Bates DV. 1977. Respiratory Effects of Exposure to Ozone Plus Sulfur Dioxide in Southern Californians and Eastern Canadians. Am. Ind. Hyg. Assoc. J. 38:696-706

Bethel RA, Epstein J, Sheppard D, Nadel JA, Boushey HA. 1983a. Sulfur dioxide induced bronchoconstriction in freely breathing, exercising, asthmatic subjects. Am. Rev. Respir. Dis. 128(6):987-990

Bethel RA, Erle DJ, Epstein J, Sheppard D, Nadel JA, Boushey HA. 1983b. Effect of exercise rate and route of inhalation on sulfur dioxide-induced bronchoconstriction in asthmatic subjects. Am. Rev. Respir. Dis. 128(4):592-596

Bethel R.A, Sheppard D, Epstein J, Tam E, Nadel JA, Boushey HA. 1984. Interaction of sulfur dioxide and dry cold air in causing bronchoconstriction in asthmatic subjects. J Appl Physiol Respirat. Environ. Exercise. Physiol. 57:419-423

Bethel RA, Sheppard D, Geffroy B, Tam E, Nadel JA, Boushey HA. 1985. Effect of 0.25 ppm sulfur dioxide on airway resistance in freely breathing, heavily exercising, asthmatic subjects. Am. Rev. Respir. Dis. 131(4):659-661

Devalia JL, Rusznak C, Herdman MJ, Trigg CJ, Davies RJ, Tarraf H. 1994. Effect of nitrogen dioxide and sulfur dioxide on airway response of mild asthmatic patients to allergen inhalation. Lancet. 344:1668-1671

Gong H, Lachenburch PA, Harber P, Linn WS. 1995. Comparative short-term health responses to sulfur dioxide exposure and other common stresses in a panel of asthmatics. Toxicol. Ind. Health 11(5):467-487

Gong H Jr, Linn WS, Shamoo DA, Anderson KR, Nugent CA, Clark KW, Lin AE. 1996. Effect of inhaled salmeterol on sulfur dioxide-induced bronchoconstriction in asthmatic subjects. Chest. 110(5):1229-1235

Gong H Jr, Linn WS, Terrell SL, Anderson KR, Clark KW. 2001. Anti-inflammatory and lung function effects of montelukast in asthmatic volunteers exposed to sulfur dioxide. Chest. 119(2):402-408

Jaeger MJ, Tribble D, Wittig HJ. 1979. Effect of 0.5 ppm sulfur dioxide on the respiratory function of normal and asthmatic subjects. Lung. 156:119-127

Jorres R, Magnussen H. 1990. Airways response of asthmatics after a 30 min exposure, at resting ventilation to 0.25 ppm NO_2_ or 0.5 ppm SO_2_. Eur. Respir. J. 3(2):132-137

Hackney JD, Linn WS, Bailey RM, Spier CE, Valencia LM. 1984. Time course of exercise-induced bronchoconstriction in asthmatics exposed to sulfur dioxide. Environ. Res. 34(2):321-327

Heath SK, Koenig JQ, Morgan MS, Checkoway H, Hanley QS, Rebolledo V. 1994. Effects of sulfur dioxide exposure on African-American and Caucasian asthmatics. Environ. Res. 66(1):1-11

Horstman DH, Seal E, Folinsbee LJ, Ives P, Roger J. 1988. The relationship between exposure duration and sulfur dioxide-induced bronchoconstriction in asthmatic subjects. Am. Ind. Hyg. Assoc. J. 49:38-47

Kehrl R, Roger LJ, Hazucha MJ, Horstman DH. 1983. Pulmonary responses of young male adult asthmatics to SO_2_ with moderate exercise. Am. Rev. Resp. Dis. 127:A160

Kehrl HR, Roger LJ, Hazucha MJ, Horstman DH. 1987. Differing response of asthmatics to sulfur dioxide exposure with continuous and intermittent exercise. Am. Rev. Respir. Dis. 135(2):350-355

Kirkpatrick MB, Sheppard D, Nadel JA, Boushey HA. 1982. Effect of the oronasal breathing route on sulfur dioxide-induced bronchoconstriction in exercising asthmatic subjects. Am Rev Respir Dis. 125(6):627-631

Koenig JQ, Pierson WE, Frank R. 1980. Acute effects of inhaled sulfur dioxide plus sodium chloride droplet aerosol on pulmonary function in asthmatic adolescents. Environ. Res. 22(1):145-153

Koenig JQ, Pierson WE, Horike M, Frank R. 1981. Effects of sulfur dioxide plus NaCl aerosol combined with moderate exercise on pulmonary function in asthmatic adolescents. Environ. Res. 25:340-348

Koenig JQ, Pierson WE, Horike M, Frank R. 1983. A comparison of the pulmonary effects of 0.5 ppm versus 1.0 ppm sulfur dioxide plus sodium chloride droplets in asthmatic adolescents. J. Toxicol. Environ. Health 11(1):129-139

Koenig JQ, Morgan MS, Horike M, Pierson WE. 1985. The effects of sulfur oxides on nasal and lung function in adolescents with extrinsic asthma. J. Allergy. Clin. Immunol. 76(6):813-818

Koenig JQ, Marshall SG, Horike M, Shapiro GG, Furukawa CT, Bierman CW, Pierson WE. 1987. The effects of albuterol on sulfur dioxide-induced bronchoconstriction in allergic adolescents. J. Allergy. Clin. Immunol. 79(1):54-58

[Koenig JQ](http://www.ncbi.nlm.nih.gov/pubmed?term=%22Koenig%20JQ%22%5BAuthor%5D), [Marshall SG](http://www.ncbi.nlm.nih.gov/pubmed?term=%22Marshall%20SG%22%5BAuthor%5D), [van Belle G](http://www.ncbi.nlm.nih.gov/pubmed?term=%22van%20Belle%20G%22%5BAuthor%5D), [McManus MS](http://www.ncbi.nlm.nih.gov/pubmed?term=%22McManus%20MS%22%5BAuthor%5D), [Bierman CW](http://www.ncbi.nlm.nih.gov/pubmed?term=%22Bierman%20CW%22%5BAuthor%5D), [Shapiro GG](http://www.ncbi.nlm.nih.gov/pubmed?term=%22Shapiro%20GG%22%5BAuthor%5D), [Furukawa CT](http://www.ncbi.nlm.nih.gov/pubmed?term=%22Furukawa%20CT%22%5BAuthor%5D), [Pierson WE](http://www.ncbi.nlm.nih.gov/pubmed?term=%22Pierson%20WE%22%5BAuthor%5D). 1988. Therapeutic range cromolyn dose-response inhibition and complete obliteration of SO_2_-induced bronchoconstriction in atopic adolescents. [J. Allergy. Clin. Immunol.](http://www.ncbi.nlm.nih.gov/pubmed/3131405) 81(5 Pt 1):897-901

Koenig JQ, Covert DS, Pierson WE. 1989. Effects of inhalation of acidic compounds on pulmonary function in allergic adolescent subjects, Environ. Health. Perspect. 79:173-178

Koenig JQ, Covert DS, Hanley QS, Van Belle G, Pierson WE. 1990. Prior exposure to ozone potentiates subsequent responses to sulfur dioxide in adolescent asthmatic subjects. Am. Rev. Respir. Dis. 141:377-380

Koenig JQ, Dumler K, Rebolledo V, Williams PV, Pierson WE. 1992. Theophylline mitigates the bronchoconstrictor effects of sulfur dioxide in subjects with asthma. J. Allergy. Clin. Immunol. 89(4):789–794

Lazarus SC, Wong HH, Watts MJ, Boushey HA, Lavins BJ, Minkwitz MC. 1997. The leukotriene receptor antagonist zafirlukast inhibits sulfur dioxide-induced bronchoconstriction in patients with asthma. Am. J. Respir. Crit. Care. Med. 156:1725-1730

Linn WS, Venet TG, Shamoo DA, Valencia LM, Anzar UT, Spier CE, Hackney JD. 1977. Respiratory effects of sulfur dioxide in heavily exercising asthmatics. Am. Ind. Hyg. Assoc. J. 38:696-706

Linn WS, Bailey RM, Shamoo DA, Venet TG, Wightman LH, Hackney JD. 1982. Respiratory responses of young adult asthmatics to sulfur dioxide exposure under simulated ambient conditions. Environ. Res. 29:220-232

Linn WS, Shamoo DA, Spier CE, Valencia LM, Anzar UT, Venet TG, Hackney JD. 1983a. Respiratory effects of 0.75 ppm sulfur dioxide in exercising asthmatics: Influence of upper-respiratory defenses. Environ. Res. 30(2):340-348

Linn WS, Venet TG, Shamoo DA, Valencia LM, Anzar UT, Spier CE, Hackney JD. 1983b. Respiratory effects of sulfur dioxide in heavily exercising asthmatics: A dose-response study. Am. Rev. Respir. Dis. 127(3):278-283

Linn WS, Avol EL, Shamoo DA, Venet TG, Anderson KR, Whynot JD, Hackney JD. 1984a. Asthmatics’ response to 6-hr sulfur dioxide exposures on two successive days. Arch. Environ. Health 39(4):313-319

Linn WS, Shamoo DA, Venet TG, Bailey RM, Wightman LH, Hackney JD. 1984b. Comparative effects of sulfur dioxide exposures at 5°C and 22°C in exercising asthmatics. Am. Rev. Respir. Dis. 129:234-239

Linn WS, Shamoo DA, Venet TG, Spier CE, Valencia LM, Anzar UT, Hackney JD. 1984c. Combined effect of sulfur dioxide and cold in exercising asthmatics. Arch. Environ. Health. 39:339-346

Linn WS, Avol EL, Shamoo DA, Peng RC, Spier CE, Smith MN, Hackney JD. 1988. Effect of metaproterenol sulfate on mild asthmatics’ response to sulfur dioxide exposure and exercise. Arch. Environ. Health. 43:399-406

Linn WS, Shamoo DA, Peng RC, Clark KW, Avol EL, Hackney JD. 1990. Responses to sulfur dioxide and exercise by medication-dependent asthmatics: Effect of varying medication levels. Arch. Environ. Health. 45:24-30

Linn WS, Shamoo DA, Anderson KR, Whynot JD, Avol EL, Hackney JD. 1985. Effects of heat and humidity on the responses of exercising asthmatics to sulfur dioxide exposure. Am. Rev. Respir. Dis. 131(2):221-225

Linn WS, Avol EL, Peng R, Shamoo DA, Hackney JD. 1987. Replicated dose-response study of sulfur dioxide effects in normal, atopic, and asthmatic volunteers. Am. Rev. Respir. Dis. 136:1127-1134

McManus MS, Koenig JQ, Altman LC, Pierson WE. 1989. Pulmonary effects of sulfur dioxide exposure and ipratropium bromide pretreatment in adults with nonallergic asthma. J. Allergy. Clin. Immunol. 83(3):619-626

Magnussen H, Jorres R, Wagner HM, von Nieding, G. 1990. Relationship between the airway response to inhaled sulfur dioxide, isocapnic hyperventilation, and histamine in asthmatic subjects. Int. Arch. Occup. Environ. Health 62(7):485-491

Roger LJ, Kehrl HR, Hazucha M, Horstman DH. 1985. Bronchoconstriction in asthmatics exposed to sulfur dioxide during repeated exercise. J. Appl. Physiol. 59(3):784-791

Sheppard D, Wong WS, Uehara CF, Nadel JA, Boushey HA. 1980. Lower threshold and greater bronchomotor responsiveness of asthmatic subjects to sulfur dioxide. Am. Rev. Resp. Dis. 122:873-878

[Sheppard D](http://www.ncbi.nlm.nih.gov/pubmed?term=%22Sheppard%20D%22%5BAuthor%5D), [Saisho A](http://www.ncbi.nlm.nih.gov/pubmed?term=%22Saisho%20A%22%5BAuthor%5D), [Nadel JA](http://www.ncbi.nlm.nih.gov/pubmed?term=%22Nadel%20JA%22%5BAuthor%5D), [Boushey HA](http://www.ncbi.nlm.nih.gov/pubmed?term=%22Boushey%20HA%22%5BAuthor%5D). 1981a. Exercise increases sulfur dioxide-induced bronchoconstriction in asthmatic subjects. [Am. Rev. Respir. Dis.](http://www.ncbi.nlm.nih.gov/pubmed/7235370) 123(5):486-491.

[Sheppard D](http://www.ncbi.nlm.nih.gov/pubmed?term=%22Sheppard%20D%22%5BAuthor%5D), [Nadel JA](http://www.ncbi.nlm.nih.gov/pubmed?term=%22Nadel%20JA%22%5BAuthor%5D), [Boushey HA](http://www.ncbi.nlm.nih.gov/pubmed?term=%22Boushey%20HA%22%5BAuthor%5D). 1981b. Inhibition of sulfur dioxide-induced bronchoconstriction by disodium cromoglycate in asthmatic subjects. [Am. Rev. Respir. Dis.](http://www.ncbi.nlm.nih.gov/pubmed/6792956) 124(3):257-259

Sheppard D, Epstein J, Bethel RA, Nadel JA, Boushey HA. 1983. Tolerance to sulfur dioxide-induced bronchoconstriction in subjects with asthma. Environ. Res. 30(2):412-419

Snashall PD, Baldwin C. 1982. Mechanisms of sulfur dioxide induced bronchoconstriction in normal and asthmatic man. Thorax. 37:118-123

Tam EK, Liu J, Bigby BG, Boushey HA. 1988. Sulfur dioxide does not acutely increase nasal symptoms or nasal resistance in subjects with rhinitis or in subjects with bronchial responsiveness to sulfur dioxide. Am Rev Respir Dis. Dec;138(6):1559-1564. Erratum in: Am. Rev. Respir. Dis. 139(6), 1579

Trenga CA, Koenig JQ, Williams PV. 1999. Sulphur dioxide sensitivity and plasma antioxidants in adult subjects with asthma. Occup. Environ. Med. 56(8):544-547

Tunnicliffe WS, Hilton MF, Harrison RM, Ayres JG. 2001. The effect of sulphur dioxide exposure on indices of heart rate **variability** in normal and asthmatic adults. Eur. Respir. J. 17:604-608

**Same data presented in Tunnicliffe et al. 2001:** Tunnicliffe WS, Harrison RM, Kelly FJ, Duster C, Ayers JG. 2003. The effect of sulphurous air pollutant exposure on symptoms, lung function, exhaled nitric oxide, and nasal epithelial lining fluid antioxidant concentration in normal and asthmatic adults. Occup. Environ. Med. 60:15

Wiebicke W, Jörres R, Magnussen H. 1990. Comparison of the effects of inhaled corticosteroids on the airway response to histamine, methacholine, hyperventilation, and sulfur dioxide in subjects with asthma. J. Allergy. Clin. Immunol. 86(6 Pt 1):915-923

Winterton DL, Kaufman J, Keener CV, Quigley S, Farin FM, Williams PV, Koenig JQ. 2001. [Genetic polymorphisms as biomarkers of sensitivity to inhaled sulfur dioxide in subjects with asthma.](http://www.ncbi.nlm.nih.gov/pubmed/11258696) Ann. Allergy. Asthma. Immunol. 86(2):232-238

[Witek TJ Jr](http://www.ncbi.nlm.nih.gov/pubmed?term=%22Witek%20TJ%20Jr%22%5BAuthor%5D), Schachter EN. 1985a. Airway responses to sulfur dioxide and methacholine in asthmatics. [J. Occup. Med.](http://www.ncbi.nlm.nih.gov/pubmed/3998877) 27(4):265-268

Witek TJ, Schachter EN, Beck GJ, Cain W, Colice G, Leaderer BP. 1985b. Respiratory symptoms associated with sulfur dioxide exposure Int. Arch. Occup. Environ. Health, 55(2):179-183

**Same data presented in Witek 1985b:** Schachter EN, Witek TJ, Beck GJ, Hosein HB, Colice G, Leaderer BP, Cain W. 1984. Airway effects of low concentrations of sulfur dioxide: Dose response characteristics. Arch. Environ. Health 39(1):34-42

Witek TJ, Schacter EN, Tosun T, Leaderer BP, Beck GJ. 1986. Controlled human studies on the pulmonary effects of indoor air pollution: experiences with sulfur dioxide and formaldehyde. Environ. Intl. 12:129-135

**SULPHURIC ACID**

Anderson KR, Avol EL, Edwards SA, Shamoo DA, Peng R-C, Linn WS, Hackney JD. 1992. Controlled exposures of volunteers to respirable carbon and sulfuric acid. J. Air. Waste. Manage. Assoc. 42:770-776

[Aris R](http://www.ncbi.nlm.nih.gov/pubmed?term=%22Aris%20R%22%5BAuthor%5D), [Christian D](http://www.ncbi.nlm.nih.gov/pubmed?term=%22Christian%20D%22%5BAuthor%5D), [Sheppard D](http://www.ncbi.nlm.nih.gov/pubmed?term=%22Sheppard%20D%22%5BAuthor%5D), [Balmes JR](http://www.ncbi.nlm.nih.gov/pubmed?term=%22Balmes%20JR%22%5BAuthor%5D). 1990. Acid fog-induced bronchoconstriction. The role of hydroxymethanesulfonic acid. [Am. Rev. Respir. Dis.](http://www.ncbi.nlm.nih.gov/pubmed/2155554) 141(3):546-551

Aris R, Christian D, Sheppard D, Balmes JR. 1991. Lack of bronchoconstrictor response to sulfuric acid aerosols and fogs. Am. Rev. Respir. Dis. 143:744-750

Avol EL, Jones MP, Bailey RM, Chang NM, Kleinman MT, Linn WS, Bell KA, Hackney JD. 1979. Controlled exposures of human volunteers to sulfate aerosols. Health effects and aerosol characterization. Am. Rev. Respir. Dis. 120(2):319-327.

Avol EL, Linn WS, Whiteman JD, Whynot JD, Anderson KR, Hackney JD. 1988a. Short-term respiratory effects of sulfuric acid in fog: a laboratory study of healthy and asthmatic volunteers. JAPCA. 38:258-263

Avol EL, Linn WS, Whynot JD, Anderson KR, Shamoo DA, Valencia LM, Little DE, Hackney JD. 1988b. Respiratory dose-response study of normal and asthmatic volunteers exposed to sulfuric acid aerosol in the sub-micrometer size range. Toxicol. Ind. Health. 4(2):173-184

Avol EL, Linn WS, Shamoo DA, Anderson KR, Peng R-C, Hackney JD. 1990. Respiratory responses of young asthmatic volunteers in controlled exposures to sulfuric acid aerosol. Am. Rev. Respir. Dis. 142:343-348

Bauer MA, Utell MJ, Speers DM, Gibbs FR, Morrow PE. 1988. Effects of near ambient levels of sulfuric acid aerosol on lung function in exercising subjects with asthma and COPD. Am. Rev. Respir. Dis. 137:167

Frampton MW, Morrow PE, Cox C, Levi PC, Condemi JJ, Speers D, Gibb FR, Utell MJ. 1995. Sulfuric acid aerosol followed by ozone exposure in healthy and asthmatic subjects. Environ. Res. 69:1-14

Hackney JD, Linn WS, Bell KA. 1978. Experimental Studies of the Human Health Effects of Sulfur Oxides. Bull. N. Y. Acad. Med. 54(11):1177–1185

Hanley QS, Koenig JQ, Larson TV, Anderson TL, Van Belle G, Rebolledo V, Covert DS, Pierson WE. 1992. Response of young asthmatic patients to inhaled sulfuric acid. Am. Rev. Respir. Dis. 145:326-331

Koenig JQ, Pierson WE, Horike M. 1983. The effects of inhaled sulfuric acid on pulmonary function in adolescent asthmatics. Am. Rev. Respir. Dis. 128:221-225

Koenig JQ, Morgan MS, Horike M, Pierson WE. 1985. The effects of sulfur oxides on nasal and lung function in adolescents with extrinsic asthma. Allergy. Clin. Immunol. 76, 813-818

Koenig JQ, Covert DS, Pierson WE. 1989. Effects of inhalation of acidic compounds on pulmonary function in allergic adolescent subjects. Environ. Health. Persp. 79:173-178

Koenig JQ, Covert DS, Larson TV, Pierson WE. 1992a. The effect of duration of exposure on sulfuric acid-induced pulmonary function changes in asthmatic adolescent subjects: a dose-response study. Toxicol. Indust. Health. 8(5):285-296

**Same data presented in Koenig et al. 1992a:** Koenig JQ, Covert DS, Larson TV, Pierson WE. 1992b. The effect of duration of exposure on sulfuric acid-induced pulmonary function changes in asthmatic adolescent subjects: a dose-response study. Am. Rev. Respir. Dis 145 (4) Part 2:A428

Koenig JQ, Dumler K, Rebolledo V, Williams PV, Pierson WE. 1993. Respiratory effects of inhaled sulfuric acid on senior asthmatics and nonasthmatics. Arch. Environ. Health. 48(3):171-175

Leduc D, Fally S, de Vuyst P, Wollast R, Yernault J-C. 1995. Acute exposure to realistic acid fog: effects on respiratory function and airway responsiveness in asthmatics. Environ. Res. 71:89-98

Linn WS, Avol EL, Shamoo DA, Whynot JD, Anderson KR, Hackney JD. 1985. Respiratory responses of asthmatic volunteers in controlled exposures to sulfuric acid aerosols. Am. Rev. Respir. Dis, 131 (4), Suppl:A201

Linn WS, Avol EL, Shamoo DA, Whynot JD, Anderson KR, Hackney JD. 1986a. Respiratory responses of exercising asthmatic volunteers exposed to sulfuric acid aerosol. J. Air Poll. Contr. Assoc. 36(12):1323-1328

Linn WS, Avol EL, Shamoo DA, Anderson KR, Whynot JD, Hackney JD. 1986b. Respiratory effects of “acid fog” exposure in normal and asthmatic volunteers. Am. Rev. Respir. Dis, 133 (4):Suppl:A214

Linn WS, Avol EL, Shamoo DA, Anderson KR, Peng R-C, Hackney JD. 1990. Respiratory response of young asthmatics to sulfuric acid aerosol. Am. Rev. Respir. Dis 141(4)Part 2: A74

Linn WS, Shamoo DA, Anderson KR, Peng R-C, Avol EL, Hackney JD. 1994. Effects of prolonged, repeated exposure to ozone, sulfuric acid, and their combination in healthy and asthmatic volunteers. Am. J. Respir. Crit. Care. Med. 150:431-440

Sackner MA, Ford D, Fernandez J, Cipley J, Perez D, Kwoka M, Reinhart M, Michaelson ED, Schreck R, Wanner A. 1978. Effects of sulfuric acid aerosol on cardiopulmonary function of dogs, sheep and humans. Am. Rev. Resp. Dis. 118:497

Spektor DM, Leikauf GD, Albert RE, Lippmann M. 1985. Effects of sub micrometer sulfuric acid aerosols on mucociliary transport and respiratory mechanics in asymptomatic asthmatics. Environ. Res. 37(1):174-191

Tunncliffe WS, Evans DE, Mark D, Harrison RM, Ayres JG. 2001. The effect of exposure to sulphuric acid on the early asthmatic response to inhaled grass pollen allergen. Eur. Respir. J. 18:640-646

Tunnicliffe WS, Harrison RM, Kelly FJ, Dunster C, Ayres JG. 2003. The effect of sulphurous air pollutant exposures on symptoms, lung function, exhaled nitric oxide, and nasal epithelial lining fluid antioxidant concentrations in normal and asthmatic adults. Occup. Environ. Med. 60:e15

Utell MJ, Morrow PE, Hyde RW. 1982. Comparison of Normal and Asthmatic Subjects Responses to Sulfate Pollutant Aerosols. Ann. Occup. Hyg. 26:691-697

Utell MJ, Morrow PE, Speers DM, Darling J, Hyde RW. 1983. Airway responses to sulfate and sulfuric acid aerosols in asthmatics. An exposure-response relationship. Am. Rev. Respir. Dis. 128:444-450

Utell MJ, Morrow PE, Hyde RW. 1984. Airway reactivity to sulfate and sulfuric acid aerosols in normal and asthmatic subjects. J. Air. Pollut. Control. Assoc. 34:931-935

Utell MJ, Morrow PE, Hyde RW, Cox C, Schreck RM. 1988. Comparison of responses and deposition following human exposures via oral or nasal inhalation of sulphuric acid aerosols. Ann. Occup. Hyg. 32:267-272

Utell MJ, Mariglio JA, Morrow PE, Gibb FR, Speers DM. 1989. Effects of inhaled acid aerosols on respiratory function: the role of endogenous ammonia. J. Aerosol. Med. 2(2):141-147

Utell MJ, Frampton MW, Morrow PE. 1991. Air pollution and asthma: clinical studies with sulfuric acid aerosols. Allergy. Proc. 12:385-388

Utell MJ, Frampton MW, Morrow PE, Cox C, Levy PC, Speers DM, Gibb FR. 1994. Oxidant and acid aerosol exposure in healthy subjects and subjects with asthma. Part II: Effects of sequential sulfuric acid and ozone exposures on the pulmonary function of healthy subjects and subjects with asthma. Res. Rep. Health Eff. Inst. 70:37-93

**4-METHYL-M-PHENYLENE**

Baur X. 1985. Isocyanate hypersensitivity. Final report to the International Isocyanate Institute III File No. 10349; III Project E-AB-19, Munich, Germany.
